# New Dimethylpyridine-3-Carboxamide Derivatives as MMP-13 Inhibitors with Anticancer Activity

**DOI:** 10.3390/molecules30244662

**Published:** 2025-12-05

**Authors:** Remigiusz Płaczek, Tomasz Janek, Małgorzata Strzelecka, Aleksandra Kotynia, Piotr Świątek, Żaneta Czyżnikowska

**Affiliations:** 1 Department of Basic Chemical Sciences, Faculty of Pharmacy, Wroclaw Medical University, Borowska 211, 50-556 Wroclaw, Poland; aleksandra.kotynia@umw.edu.pl; 2 Department of Biotechnology and Food Microbiology, Faculty of Biotechnology and Food Science, Wroclaw University of Environmental and Life Sciences, Chełmońskiego 37, 51-630 Wroclaw, Poland; 3 Department of Medicinal Chemistry, Faculty of Pharmacy, Wroclaw Medical University, Borowska 211, 50-556 Wroclaw, Poland; malgorzata.strzelecka@umw.edu.pl (M.S.); piotr.swiatek@umw.edu.pl (P.Ś.)

**Keywords:** dimethylpyridine-3-carboxamide derivatives, MMP-13 inhibitors, anticancer activity

## Abstract

A series of dimethylpyridine-3-carboxamide derivatives was designed as potential, selective, non-zinc chelating inhibitors of matrix metalloproteinase 13 (MMP-13), and subsequently synthesized. The identity of the obtained compounds was confirmed by FT-IR, ^1^H/^13^C NMR, and HR-MS methods. Fluorescence spectroscopy was applied to study the interaction of synthesized compounds with human serum albumin, providing insight into their potential transport properties in plasma. In parallel, the electronic properties and reactivity parameters relevant to enzyme binding of the designed molecules were analyzed using density functional theory. Molecular docking and molecular dynamics simulations revealed the compounds to interact preferentially and stably within the S_1_ pocket of MMP-13 via hydrogen bonds and π-stacking interactions. The calculated binding free energy confirmed the stability and persistence of the complexes during simulation, indicating a strong and specific recognition pattern. On the other hand, their affinity towards MMP-8 was considerably weaker, which is consistent with the predicted selectivity profile. In addition, the biological evaluation confirmed MMP-13 inhibition. Finally, in vitro tests revealed their cytotoxic activity against cancer cell lines.

## 1. Introduction

Matrix metalloproteinases (MMPs) are zinc-dependent enzymes that are responsible for the degradation of extracellular matrix (ECM) components. This process is a critical stage in the physiological remodeling of tissue. The activity of MMPs is strictly regulated by tissue inhibitors of metalloproteinases (TIMPs). However, during disease states, the balance between MMPs and TIMPs is disrupted, leading to pathological MMP activity. This effect has been reported in diseases such as rheumatoid arthritis (RA) and osteoarthritis (OA) [1,2]. Among MMPs, collagenase-3 (MMP-13) has specific potency in the degradation of type II collagen. In inflammatory joint conditions, chondrocytes significantly upregulate MMP-13 synthesis. Reduced TIMP regulation leads to an accumulation of collagen degradation products, which, in turn, have been shown to overwhelm phagocytic mechanisms and promote inflammation. Persistent autoimmune inflammation, a key feature of RA, has been shown to accelerate cartilage destruction, joint deterioration, and disability. Current RA/OA therapeutics, including methotrexate, leflunomide, anti-cytokine biologics, sulfasalazine, and COX inhibitors, have been shown to reduce symptoms and autoimmune pathways associated with the disease. However, these pharmaceuticals do not directly inhibit MMP-13, which remains a contributing factor to joint damage and chronic disease progression [3]. Moreover, recent findings suggest that MMP-13 plays an important role in cancer progression and metastasis. Overexpression of MMP-13 correlates with tumor aggressiveness and poor prognosis in breast, colorectal, prostate, bladder, and lung cancers. Its broad ECM-degrading capability has been demonstrated to promote invasion and angiogenesis. Additionally, MMP-13 may activate pro-MMP-9, thus facilitating vascular intravasation and extravasation by cancer cells. Specifically, in melanoma, high MMP-13 levels are associated with metastasis and reduced patient survival. Furthermore, MMP-13 expression in melanoma cells has been linked to increased migratory activity and epithelial–mesenchymal transition marker expression [4,5]. In addition, MMP-13-mediated proteolysis of laminin-5 has been shown to enhance tumor invasiveness, though it concurrently disrupts vasculogenic mimicry [6]. Similarly, in colorectal cancer MMP-13 plays an important role in tumor progression [7]. It has been demonstrated that its overexpression in HT-29 cells promotes invasion angiogenesis and matrix remodeling in models of colon cancer, whereas the silencing of MMP-13 significantly reduces tumor extravasation in the liver in vivo [8,9]. In this context, there is evidence that inhibition of MMP-13 may promote cancer cell death by inducing ferroptosis [10].

**Figure 1:**
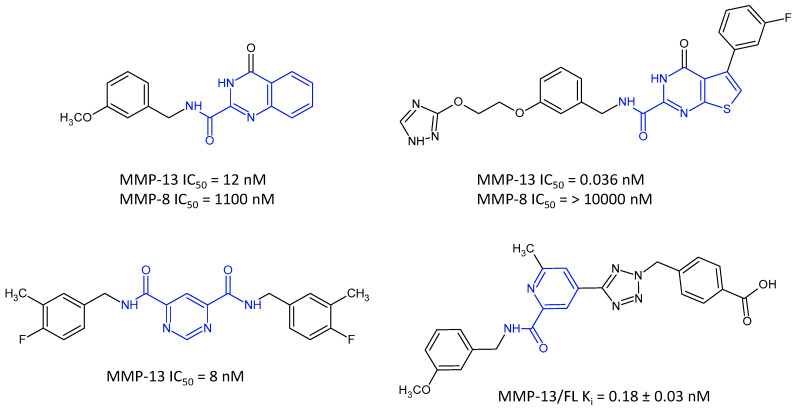
Selected non-zinc chelating MMP-13 inhibitors.

In the early stages of research, the development of MMP-13 inhibitors focused on zinc-chelating compounds targeting the catalytic site. Despite their potency, these agents exhibited a lack of selectivity due to the similar nature of the catalytic zinc-binding motif across MMP family members. Non-selectivity of compounds results in adverse outcomes, including soft-tissue fibromas, musculoskeletal syndrome (MSS), and systemic toxicity [11]. Consequently, research shifted towards the design of non-zinc-binding inhibitors targeting the S_1_′ specificity pocket, unique to MMP-13 [12]. A number of these compounds, representing various derivatives of heterocyclic scaffold bearing carboxamide or dicarboxamide groups, have demonstrated high potency, selectivity, and favorable biological profiles both in vitro and in vivo (Figure 1). These were quinazoline acid derivatives without joint fibroplasia side effects, and *N*-Isopropoxysulfonamide based hydroxamates derivatives [13,14]. In addition, carboxylic acid derivatives, pyrimidine-2-carboxamide and hybrid structures of quinazoline with a triazole group were found as selective zinc-binding inhibitors of MMP-13 [15,16,17]. Moreover, strong selectivity over other MMPs has also been reported for quinazolinone and pyrido[3,4-*d*]pyrimidin-4-one derivatives as well as for (pyridine-4-yl)-2*H*-tetrazole [18,19]. Among these, orally bioavailable compounds showed protective effects in cartilage damage models, although clinical translation has been hindered by poor solubility and pharmacokinetics.

In our recently published work, we have shown that dimetylpyridine-3-carboxamides have a strong cytotoxic effect on cancer cells and are safer for normal cells than the reference drugs [20,21]. Importantly, MTT assays demonstrated a clearly favorable therapeutic index, with IC_50_ values of 3–10 µM for cancer cell lines (A549, MCF-7, LoVo, LoVo/Dx) and 50–130 µM for normal fibroblasts and epithelial cells (NHDF, V79, VERO) [22]. Thus, selectivity ratio ranged from 1.3 to 27 fold depending on the structure. Furthermore, compounds derived from dimethylpyridine-carboxamide scaffold also exhibited pronounced anticancer activity against melanoma, glioblastoma, and breast cancer cell lines (A375, C32, SNB-19, MCF-7/WT, MCF-7/DX), while demonstrating lower cytotoxicity towards normal keratinocytes (HaCaT) [23]. In particular, some compounds selectively reduced the viability of melanoma cells at concentrations where the effect on normal skin cells remained limited (e.g., (IC50 = 80.79 µM in A375 cells after 24 h). Our results support the notion that cancer cells are more vulnerable to compounds with this scaffold than non-malignant cells, which significantly reduces the pharmacological risk associated with systemic toxicity.

**Figure 2:**
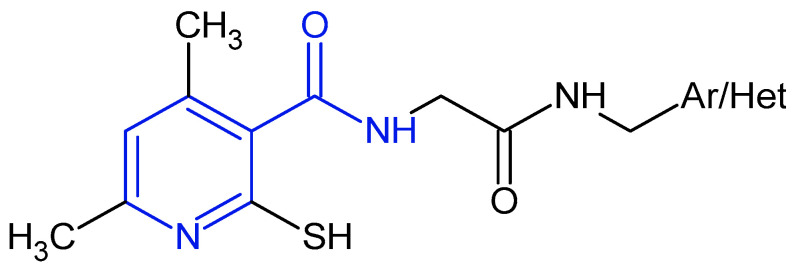
General structure of the target compounds.

In the present study, eleven newly designed and synthesized dimethylpyridine-3-carboxamide derivatives were evaluated for their multidirectional activity as potential selective MMP-13 inhibitors compared to other MMP enzymes. The general formula of the new compounds is presented in Figure 2. The key structural elements determining their biological activity include external aromatic or heterocyclic rings connected by a bridge containing two carboxamide residues. Introduction of substituents with diverse electronic properties into the aromatic rings was intended to investigate structure-activity relationships.

The electronic properties of the molecules and global reactivity descriptors were calculated to assess chemical stability and potential interaction sites. Furthermore, fluorescence spectroscopy was used to investigate the interaction of the compounds with the transport protein, human serum albumin (HSA). In silico studies were complemented by ADMET evaluation.

Due to the structural similarity exhibited by members of the MMP family, particularly MMP-8 and MMP-13, a series of detailed computational studies was conducted to investigate the selectivity of the designed ligands. Therefore, molecular docking and molecular dynamics simulations were performed not only for MMP-13, but also for MMP-8, to gain insight into binding modes and selectivity profiles. In order to confirm the ability to inhibit the selected molecular target, in vitro enzyme activity inhibition tests were performed.

As mentioned above, inhibition of MMP-13 may contribute to the inhibition of tumor progression, including processes such as invasion, angiogenesis, and metastasis. For this reason, the anticancer potential of the most promising derivatives was investigated. The ability of compounds to inhibit cancer cell viability was assessed using MTT assays.

## 2. Results and Discussion

### 2.1. Chemistry

**Scheme 1:**
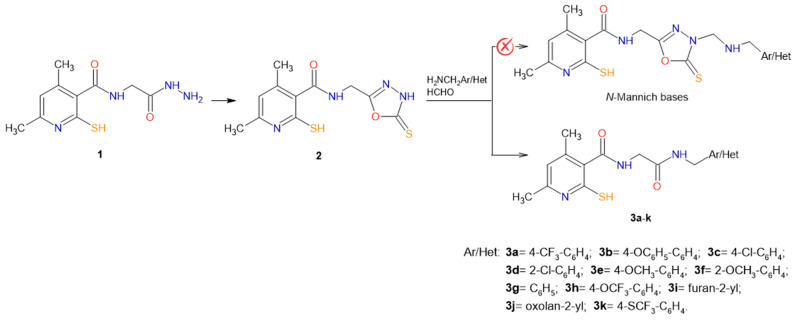
General procedure for the preparation of compounds **3a**–**k**.

The synthetic route leading to the formation of the titled derivatives is outlined in Scheme 1. The starting material, 4,6-dimethyl-*N*-(2-hydrazinyl-2-oxoethyl)-2-sulfanylpyridine-3-carboxamide **1**, and 4,6-dimethyl-*N*-[(5-sulfanylidene-4,5-dihydro-1,3,4-oxadiazol-2-yl)methyl)-2-sulfanylpyridine-3-carboxamide **2** were prepared according to the protocols published previously [20,22].

The synthetic pathway and the applied reaction conditions were intended to afford *N*-Mannich base derivatives. This approach represents a classical multicomponent reaction (MCR) previously utilized by our group to synthesize Mannich-type compounds derived from 1,3,4-oxadiazole or 1,2,4-triazole scaffolds [21,23]. In the present study, the reaction was carried out as a three-component condensation between the starting compound **2** containing an active hydrogen atom, formaldehyde, and a primary amine reagent in ethanol at room temperature. However, unexpectedly, the products obtained did not retain the oxadiazole ring within their structures. This was evidenced by the signals observed in the NMR spectra. The expected aminomethylated oxadiazoles were excluded due to the absence of methylene linker protons in the ^1^H NMR spectrum, as well as the lack of characteristic carbon signals corresponding to the oxadiazole ring in the ^13^C NMR spectrum. It was found that the 1,3,4-oxadiazole derivative **2** exhibited different reactivity toward primary amines (such as benzylamine or furfurylamine) compared to its behavior toward secondary amines (e.g., piperazine derivatives or morpholine) observed in our previous studies. This difference may be attributed to the higher basicity of primary amines relative to secondary amines, which likely promotes the ring-opening process. The instability of the 1,3,4-oxadiazole scaffold under basic conditions has been well documented in the literature [24,25].

Subsequently, efforts were directed toward optimizing the reaction conditions. These studies revealed that the presence of formaldehyde was essential for the reaction to proceed. Moreover, heating the reaction mixture to reflux in ethanol significantly accelerated the degradation of the 1,3,4-oxadiazole ring, compared to reactions conducted at room temperature. Notably, the overall yield remained unaffected by this temperature change.

The spectroscopic properties of all newly obtained derivatives were determined based on spectral data analysis, such as FT-IR, ^1^H NMR, ^13^C NMR, and HR-MS. All are summarized in the experimental section and documented in the Supplementary Data (Tables S1–S3). The FT-IR spectra of compounds **3a**–**k** exhibited absorption bands in the 1667–1617 cm^−1^ range, characteristic of the C=O stretching vibrations associated with the amide functional groups. Additionally, NH stretching bands of the CONH moiety were observed in the 3210–3188 cm^−1^ region. The ^1^H NMR spectra of final compounds revealed two doublet signals in the ranges of 3.80–3.84 ppm and 4.25–4.42 ppm, respectively, attributed to the methylene protons of the linker units. Moreover, two triplet signals of the NH protons appeared in the 8.74–8.85 ppm and 8.86–9.17 ppm regions. The aromatic region displayed signal between 6.25–7.66 ppm, with a characteristic pattern of para-substituted aromatic rings observed for compounds **3a**, **3b**, **3c**, **3h**, and **3k**. Notably, the SH proton signals were detected as singlets in the 13.47–13.52 ppm range, confirming the presence of thiol group. The ^13^C NMR spectra supported these findings, showing methylene carbon signals in the ranges of 35.67–42.79 ppm and 42.83–42.95 ppm, respectively. Aromatic carbon resonances were observed within the expected chemical shift range, with two carbonyl carbons resonating at approximately 167.27–167.45 ppm and 169.24–169.74 ppm. Moreover, mass spectral analysis was carried out, and the molecular ion peaks [M + H]^+^ were found to be in correlation with the corresponding calculated molecular mass, confirming the structure of the synthesized molecules. Additionally, the obtained data supported the formation of new derivatives with [M + Na]^+^ and [M + K]^+^ ion peaks.

### 2.2. Properties of Designed Compounds

#### 2.2.1. Reactivity Parameters

Electronic properties such as the energies of the highest occupied (E_HOMO_) and lowest unoccupied (E_LUMO_) orbitals, and HOMO LUMO gap are effective descriptors of a molecule’s capacity to exchange electrons (See Table 1 and also in Supplementary Materials see Table S4 for molecular orbitals). In a biological context, these values provide a simple way to understand reactivity patterns, which may manifest in differences in potency, likelihood of covalent system formation, redox properties, and even metabolic stability. According to the data, a less negative E_HOMO_ value (E_HOMO_ **_3a_**=−6.53 eV, E_HOMO_ **_3j_** = −6.68 eV) implies a lower ionization potential and, therefore, easier oxidation. Such compounds tend to be more reactive under oxidative conditions. EA lower value of E_LUMO_ indicates a higher electron affinity, which can correlate with stronger H-bond acceptor character of heteroatoms and better charge acceptance from protein donors. The electron-accepting character of **3k**, **3j**, and **3f** is strongest among all compounds, as shown by their low value of E_LUMO_ and high EA. In the context of interactions with MMP-13, it is expected that the compounds will readily interact with Lys/Thr/His residues in the S_1_′ pocket. A smaller HOMO-LUMO gap (resulting in lower η and higher s) indicates a softer, more polarizable species that redistributes charge more easily upon binding. This often results in higher potency, but also greater reactivity. According to results, compounds with a larger gap (**3i**—GAP = 5.17 eV; **3a**—GAP = −5.11 eV and **3h**—GAP = −5.11 eV) are harder and consequently more kinetically stable. These properties may promote selectivity and their metabolic stability. Higher electronegativity (χ), and therefore a more negative μ value, indicates a stronger tendency to accept electron density during intermolecular interactions. As demonstrated in this series, the **3j** and **3k** compounds exhibit the highest values of χ. Conversely, **3e**, **3b**, **3g**, and **3i** (χ ≈ 3.65–3.68 eV) are weaker electron acceptors. The electrophilicity index is a complex measure of the ability of compounds to accept electrons, combining chemical potential and hardness. In the designed series, ω ranges from 6.64 to 8.65 eV. The compounds with the highest electrophilicity values are **3j** (8.65 eV) and **3k** (8.13 eV), while the lowest values are observed in **3e** (6.64 eV) and **3b** (6.73 eV). Overall, differences in χ and ω may support subtle variations in the interaction potential and reactivity profile of tested molecules.

#### 2.2.2. ADMET Evaluations

To evaluate the ADMET properties of the most potent compounds (according to experimental results), the ADMETLab 3.0 platform was used. In this study, the focus was placed on several key pharmacokinetic parameters. The ADMET data for the most active derivatives (**3b**, **3c**, **3d**, and **3g**) are presented in Table 2, whereas the full data set for all designed compounds is provided in the Supplementary Materials (see Table S6).

First, Lipinski’s Rule was assessed, which considers the main physicochemical properties of drug-like molecules, including molecular weight, hydrogen bond donors and acceptors, and lipophilicity (logP). All designed compounds met these criteria, indicating favorable physicochemical characteristics and no violations of the rule. In the next step, the absorption parameters were analyzed. Subsequently, an analysis was performed on absorption-related parameters. The Caco-2 cell permeability test predicted good permeability only for compounds **3c**, **3h** and **3k**, whereas the remaining compounds showed low permeability. However, the human intestinal absorption (HIA) indicated that all compounds are expected to be absorbed at levels exceeding 30% of the administered dose, suggesting that intestinal absorption should still occur to a relevant extent. These findings are further supported by the predicted topological polar surface area (TPSA) values (71–84 Å^2^), which fall within the recommended range for orally bioavailable molecules. Such TPSA values suggest that all compounds are likely to exhibit favorable passive permeability. The predicted MDCK permeability values (−5.06–4.65 cm/s) fall within the medium permeability range, indicating that passive epithelial transport is expected to be moderate. These values remain consistent with acceptable oral absorption properties for compounds of this physicochemical class. The distribution properties were also examined. All compounds demonstrated a high degree of plasma protein binding (PPB), which will be further verified experimentally using spectroscopic methods. The predicted volume of distribution (VD_ss_ ≈ 1.0–1.6 L/kg) suggests a moderate distribution within body tissues, indicating that the compounds are likely capable of reaching the extracellular fluids but are not expected to accumulate in tissues. Moreover, metabolic stability and elimination profiles were evaluated. The predicted clearance (CL_plasma_) indicates a moderate elimination rate, no with tendency toward rapid metabolic degradation. The predicted values range approximately from 4.18 mL/min/kg to 5.80 mL/min/kg for **3b** and **5a**, respectively. This observation is consistent with the predicted half-life values (T_1/2_ ≈ 0.46–0.60), which remain within an acceptable range for small-molecule drug candidates. It also suggests that sufficient systemic exposure can be achieved to sustain interaction with the therapeutic agent. Overall, the predicted ADMET profiles demonstrate that the designed molecules possess suitable properties for further development as orally available MMP-13 inhibitors. Across all toxicity endpoints predicted by ADMETLab, four key parameters were taken into consideration. The probabilities of hERG inhibition, a measure of potential cardiotoxicity, remained low for all tested compounds. In terms of mutagenicity, compound **3b** exhibited the highest predicted AMES toxicity, whereas the remaining compounds showed reduced but still notable values. These results are consistent with the carcinogenicity predictions, as AMES mutagenicity is closely related to carcinogenic potential. Rat oral acute toxicity is one of the most important parameters for assessing the safety of new drug candidates. For all compounds in this study, the predicted probabilities of acute toxicity fell within a moderate range.

### 2.3. Molecular Docking and Molecular Dynamics

#### 2.3.1. Molecular Targets

**Figure 3:**
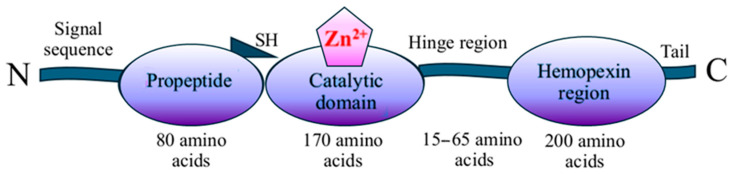
General structure of collagenases.

Collagenases belonging to the matrix metalloproteinase family are characterized by a conserved four-domain architecture (see Figure 3). The *N*-terminal propeptide, which is composed of approximately 80 residues, contains the well-conserved “cysteine switch” motif. In this motif, the thiolate group coordinates the catalytic Zn^2+^ ion. The catalytic domain contains the conserved HExxHxxGxxH motif with characteristic binding pockets (S_1_, S_2_, … and S_1_′S_2_′, …). Two catalytic Zn^2+^ ions bind to the imidazole rings of the three histidine residues (His201, His205, and His211). Furthermore, the presence of a variable-length linker (hinge) and an approximate 200-residue hemopexin-like (Hpx) domain has been observed [26].

According to Engel et al., a characteristic structural feature of MMP-13 is its deep S_1_ pocket containing the catalytic zinc ion, which constitutes the structural basis for the high selectivity of its inhibition [27]. In addition, inhibitor selectivity is found to be influenced by the nature of amino acid residue 218 and by the specificity loop (amino acid residues 244–255), which are positioned adjacent to the zinc-distal region of the S_1_′ pocket. To design the compounds with a higher selectivity profile, matrix metalloproteinase 8 (MMP-8) was selected as a second molecular target [28]. As shown, MMP-8 also belongs to the group of collagenases and is responsible for the preferential degradation of other collagen subtypes, fibronectin, and proteoglycans. Its activity is regulated by pro-inflammatory cytokines [29]. In contrast to MMP-13, the binding pocket of MMP-8 is significantly shallower and has a different character due to its amino acid composition, which makes it a valuable reference for evaluating the structural determinants of selectivity. The intermolecular interactions within the binding pockets of MMP-13 and MMP-8, which are essential for selectivity, are presented in Table 3.

An important aspect of the rational design of non-zinc chelating MMP-13 inhibitors is the incorporation of a structural moiety capable of establishing strong hydrophobic interactions with the S_1_′ pocket and its side pocket (S_1_′). Another important feature is the ability of structural elements of the inhibitor to form hydrogen bonds, especially with Lys140. In particular, functional groups with hydrogen bond donor or acceptor capacity, such as amide moieties, heteroaromatic nitrogen, or hydroxyl groups, can participate in this interaction, thereby enhancing binding affinity and selectivity.

#### 2.3.2. Molecular Docking

**Figure 4:**
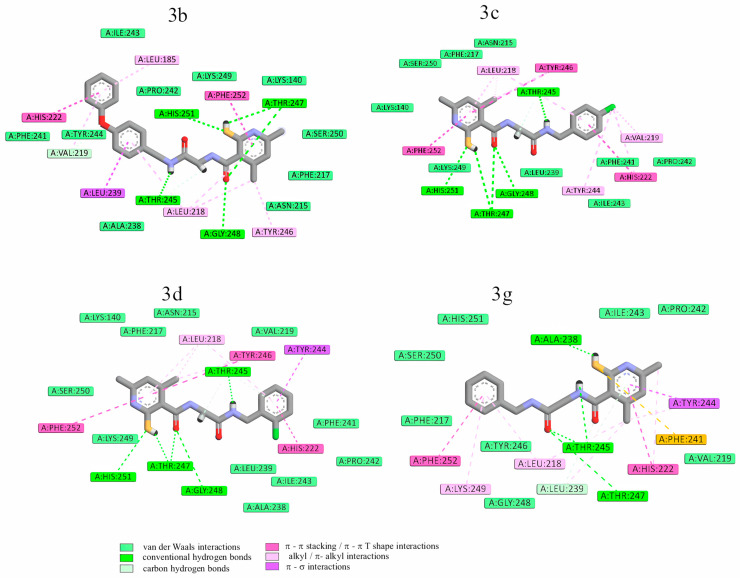
The binding mode of **3b**, **3c**, **3d**, and **3g** in the pocket of MMP-13.

Designed compounds were subsequently optimized and docked into the selected molecular targets. To propose the best binding manner of compounds, molecular docking was performed. As data revealed, all inhibitors bind to the pocket of MMP-13 and can interact with the crucial amino acid residues. The following section will present the results for the four compounds (**3b**, **3c**, **3d**, and **3g**) that exhibited the lowest binding energies and a high level of selectivity, as determined by molecular dynamics simulations. The remaining derivatives are presented in the Supplementary Table S5. As demonstrated in Figure 4, in the cases of **3b**, **3c**, and **3d**, the phenyl ring, along with its substituents, is stabilized within the S_1_′ pocket through van der Waals and π-type interactions with Pro242, Tyr244, and His222. It is noteworthy that His222, which is directly implicated in Zn^2+^ coordination, also contributes to interactions with potent inhibitors. As mentioned above, hydrogen bonds represent a crucial factor responsible for the inhibition of the enzyme activity. The three compounds under consideration form four hydrogen bonds, involving three amino acid residues. Thr245, Thr247, and Gln248. Specifically, the carbonyl and amide groups of the ligands act as hydrogen bond acceptors and donors, respectively, forming stable interactions with the side-chain amino acids. Such bonding patterns further contribute to the stabilization of the inhibitor within the active site.

**Figure 5:**
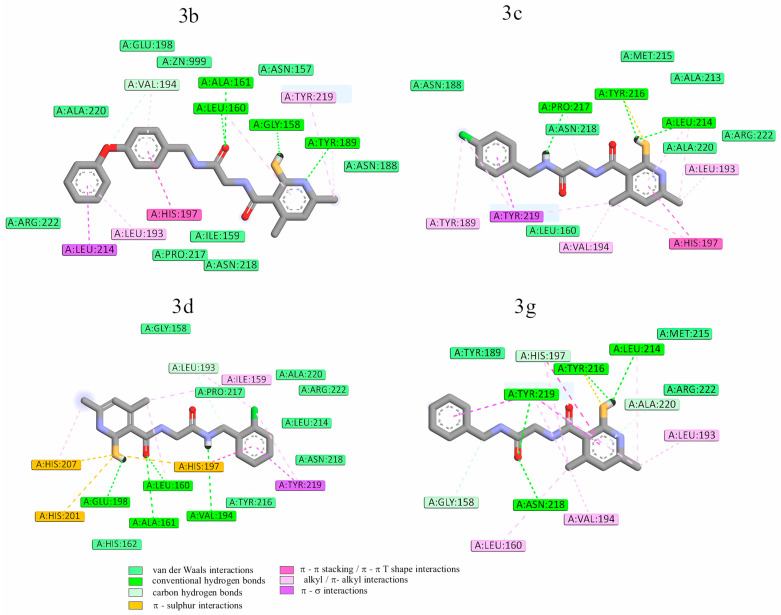
The binding mode of **3b**, **3c**, **3d**, and **3g** in the pocket of MMP-8.

In contrast, **3g** exhibits a preferential binding within the S_1_′ pocket through its 4,6-dimethyl-2-sulfanyl-pyridine-3-carboxamide moiety. In this case, the hydrogen bonds are established with Ala238 instead of Gly residues. According to molecular dynamics simulations, the designed compounds exhibited considerably weaker binding to MMP-8, as reflected by their higher free binding energy values (Section 2.3.3). Docking studies indicated that the size of the designed compounds prevents their accommodation within the MMP-8 binding pocket, thereby hindering interactions with essential amino acid residues (see Figure 5). The remaining data are presented in the Supplementary Table S5.

#### 2.3.3. Molecular Dynamic Simulations

**Figure 6:**
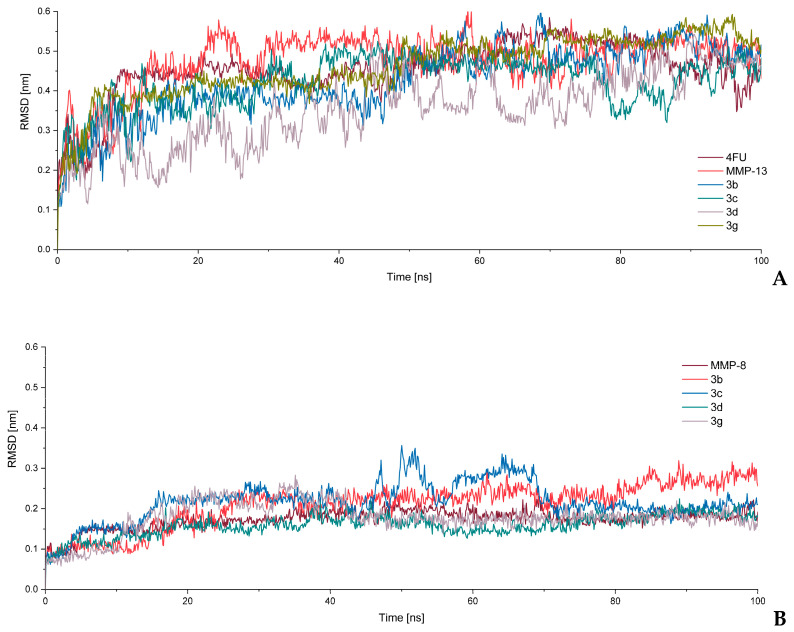
RMSD plots during 100 ns MD simulations. Backbone-to-backbone plots of protein and protein complex with designed ligands. Plots of MMP-13 (**A**) and MMP-8 (**B**).

To evaluate the affinity of the designed compound toward MMP-13 and MMP-8, we performed a series of molecular dynamics simulations. Once we obtained structures of complex ligand with metalloproteinase from molecular docking, we used them to build a system for MDs with the best position of ligand inside the active pocket of enzymes. The ligand–protein complex stability was monitored during 100 ns simulations. The RMSD plot of the backbone protein, both in the presence and absence of ligands, is shown in Figure 6A. For MMP-13 simulations, we also use the native ligand from the crystal (PDB ID: 4FU) [30] as a positive control. The plots demonstrated that all compounds reduced the fluctuation of protein during the simulations. Additionally, the constructed systems show that all complexes remained stable throughout all simulations, without significant fluctuations monitored after a few initial ns, when the system stabilizes. Importantly, for most of the simulation time, the presence of the ligand decreased the backbone fluctuations. The mean RMSD values were 4.5 Å, 4.8 Å, 4.3 Å, 4.2 Å, 3.6 Å, and 4.6 Å for 4FU, MMP-13, **3b**, **3c**, **3d**, and **3g**, respectively.

To evaluate compounds’ selectivity complex of each ligand with MMP-8 was obtained, as previously, from molecular docking, and used to build MD system positioning the ligand in the most favorable conformations. The RMSD plots of the MMP-8 backbone, in the absence and presence of ligands, are presented in Figure 6B. During the entire simulation, only minor backbone fluctuations were observed, indicating stable complex formation. However, compound **3c** demonstrated a temporary increase in RMSD amplitude of approximately 1.5 Å around the midpoint of the trajectory, which returned to a stable conformation near 70 ns, consistent with the stable state observed before 50 ns. Overall, all ligand–MMP-8 complexes demonstrated structural stability during the simulations, without pronounced deviations in RMSD values after the initial equilibration phase.

Comparative analysis of the MD simulations revealed differences in the dynamic behavior of the protein–ligand complexes, supporting the hypothesis of selective binding. To better understand those subtle differences, we used GMX_MMPBSA tool to calculate the binding free energy constructed complexes of both enzymes and ligands. Calculated values of binding free energy are shown in Table 4.

Although RMSD analysis provides valuable insights into the dynamic stability of ligand–enzyme complexes, it is not sufficient alone to determine ligand affinity. Therefore, binding free energy calculations were performed for representative frames extracted from 100 ns MD trajectories. The results revealed significant differences in the interaction energies of the designed ligands with the two MMP enzymes. Compounds optimized toward MMP-13 formed energetically more favorable complexes compared to MMP-8, indicating a clear preference for MMP-13 binding. Notably, the binding free energy values obtained for the designed inhibitors were comparable to those of the native ligand 4FU, particularly for compounds **3b** and **3c**. These findings further support the selectivity and potential effectiveness of the newly designed inhibitors.

**Figure 7:**
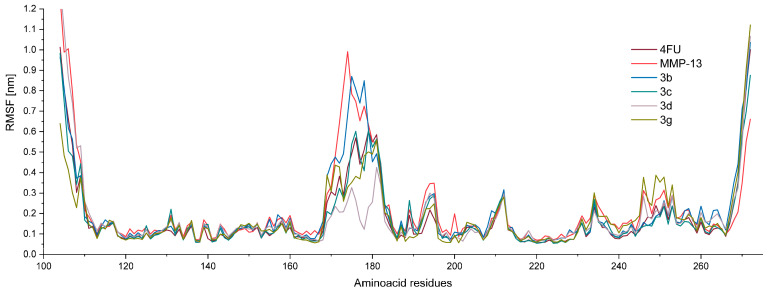
RMSF plot of MMP-13 residues in the absence and presence of designed compounds.

From MD simulations, we also obtained the RMSF values for amino acid residues in MMP-13. That shows us how changes are in the active pocket of collagenase-3. Figure 7 demonstrates that the presence of ligand significantly reduced the fluctuation of amino acid, in particular, those residues that build the active pocket of the enzyme, including: Tyr46, His222, Pro242, Tyr244, Thr245, and Thr247.

### 2.4. MMP-13 and MMP-8 Inhibitory Activity

**Figure 8:**
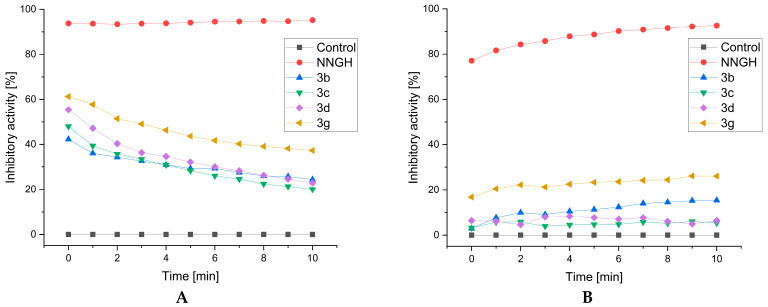
Inhibitory activity of designed compounds on MMP-13 (**A**) and MMP-8 (**B**).

The inhibitory activity of the designed compounds against MMP-13 was evaluated using a commercial fluorometric MMP-13 inhibitor assay kit (Abcam, Cambridge, UK), following the manufacturer’s protocol. Solutions of the tested compounds were prepared at concentrations ranging from 1.8 to 4.6 µM. *N*-isobutyl-*N*-(4-methoxyphenylsulfonyl)glycyl hydroxamic acid (NNGH), a broad-spectrum inhibitor of matrix metalloproteinases, was used as positive control. NNGH effectively inhibits several MMP family enzymes, including MMP-1, -3, -8, -9, -10, -12, and -13. As shown in Figure 8A, NNGH formed a stable complex with MMP-13, suppressing its activity by nearly 97%, and thus served as a positive control in the assay. All designed compounds inhibited MMP-13 within the tested concentration range, with compound **3g**, featuring a benzylamino group in the side chain, demonstrating the most pronounced inhibitory effect. In parallel, MMP-8 inhibition assays were also conducted using the same concentrations of compounds **3b**, **3c**, **3d**, and **3g**. It was evident that all tested compounds exhibited markedly weaker inhibition of MMP-8 activity. Among the four tested compounds, only compound **3g** showed slight inhibitory activity toward MMP-8, which is consistent with the results obtained from computational studies (Figure 8B).

It should be emphasized that these assays were performed as single-point screening experiments, optimized for rapid qualitative assessment rather than full kinetic characterization. Consequently, the observed differences in activity between MMP-13 and MMP-8 are indicative trends supporting the selectivity predicted by computational studies, including docking, molecular dynamics simulations, and binding free energy calculations, rather than providing definitive quantitative evidence.

### 2.5. Fluorescence Spectroscopy and UV-Vis Measurements

Albumin is the most important transport protein in plasma, binding many endogenous and exogenous compounds. One of the key steps in pharmacokinetic assessment is studying the binding of new drugs to human serum albumin (HSA). It is worth noting that only the non-protein-bound fraction of a drug is pharmacologically active, enabling it to cross biological membranes, act on receptors, and undergo metabolism [31,32,33]. Conversely, albumin-bound drugs are protected from rapid renal filtration and enzymatic degradation [34,35]. Therefore, the degree of binding influences the half-life and dosing regimen. Furthermore, knowledge of the degree of albumin binding can predict the risk of drug accumulation and toxicity [34,36].

**Figure 9:**
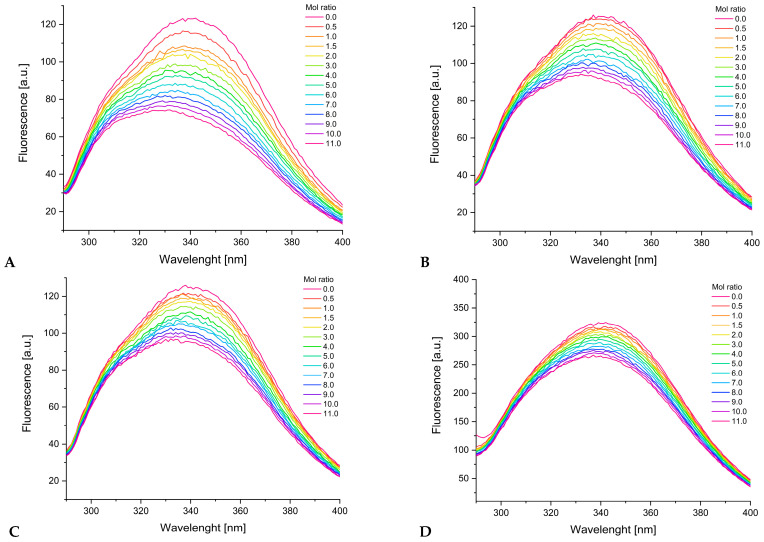
Fluorescence quenching spectra of HSA (1 × 10^−6^ mol/dm^3^) in the presence of various molar ratios of compounds **3b** (**A**), **3c** (**B**), **3d** (**C**), and **3g** (**D**) at 297 K.

In the human albumin, the amino acid residue Trp214 in subdomain IIA is of particular significance in structural studies. Subdomain IIA is responsible for binding many drugs and endogenous compounds [37]. To investigate the interactions between HSA and the tested compounds, fluorescence spectroscopy was conducted. Titration measurements were performed on the protein solution and the addition of **3b**, **3c**, **3d**, and **3g** compounds at three different temperatures: 297, 303, and 308 K. Fluorescence quenching was observed in all cases, which confirmed interaction between molecules (Figure 9). The Stern–Volmer constant with inner filter correction was applied, based on changes in absorbance and in the fluorescence intensity of excited Trp214 residues located in the protein binding pocket:

(1) $$\frac{F_{0}}{F_{corr}}=1+k_{q}\tau \left[Q\right]=1+K_{SV}[Q]$$

(2) $$F_{corr}=F_{obs}10^{\frac{\left(A_{ex}+A_{em}\right)}{2}}$$

where *F*_0_ is maximum fluorescence intensities protein without a quencher, *F_corr_* is the corrected florescence intensities, *k_q_* is the quenching rate constant of the molecules, *τ* is the average lifetime of the molecule (for biomolecules τ = 10^−8^ s [38]), [*Q*] is the concentration of quencher, *K_sv_* is the Stern-Volmer constant, *F_obs_* is the steady-state fluorescence intensity at the maximum wavelength with the quencher, *A_ex_* and *A_em_* are the absorbance at excitation and emission wavelengths, respectively. The calculated quenching parameters are presented in Table 5. The quenching rate constants (*k_q_*) are higher than 2 × 10^10^ dm^3^ mol^−1^ s^−1^; therefore, the quenching mechanism is static and supports complex formation [38,39]. Furthermore, it was demonstrated that the values of *K_SV_* and *k_q_* decrease with an increase in temperature, which also serves to confirm the static mechanism of fluorescence quenching.

The binding parameters were evaluated by a double logarithm regression curve by Equation (3):

(3) $$log\frac{F_{0}-F}{F}=\log K_{b}-n\log\left[Q\right]$$

where *F*_0_ is the maximum fluorescence intensity of a protein without a quencher, *F* is the maximum fluorescence intensity of the protein after a quencher addition, *K_b_* is the binding constant, [*Q*] is the molar concentration of the quencher, and n is the binding stoichiometry.

**Figure 10:**
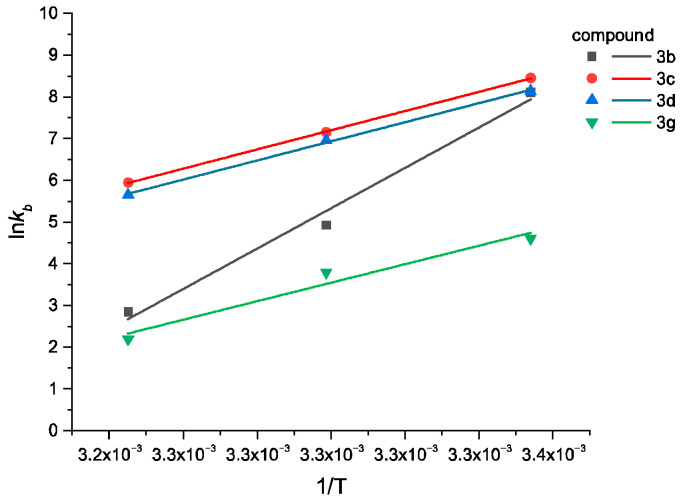
The van’t Hoff plot for the interaction of **3b**, **3c**, **3d**, and **3g** compounds with HSA.

The number of binding sites (n) heading towards one indicates that only one binding site is occupied. The compounds studied can be arranged in accordance with the value of *K_b_*, the strength of their interaction with HSA. In this ranking, **3d** is the highest ranked, followed by **3c**, **3b**, and **3g** in descending order of compatibility. Then, using Equations (4), and van’t Hoff function (5), the thermodynamic parameters of complex formation were determined and collected in Table 5, and Figure 10:

(4) $$\log K_{b}=-\frac{\Delta H^{0}}{RT}+\frac{\Delta S^{0}}{RT}$$

(5) $$\Delta G^{0}=\Delta H^{0}-T\Delta S^{0}=-RT\ln K_{b}$$

where *R* is the gas constant (8.314 J mol^−1^ K^−1^), T is temperature, *K_b_* is the binding constant, Δ*H*^0^ is enthalpy, Δ*S*^0^ is entropy, and Δ*G*^0^ is free energy change.

The complexation reaction of dimethylpyridine-3-carboxamide derivatives with HSA has been shown to occur spontaneously in experimental conditions, as evidenced by the negative Gibbs free energy. Furthermore, the negative enthalpy suggests that the process is accompanied by energy release, indicating that the interactions are energetically favorable. The complex formations may be attributed to hydrogen bonds, van der Waals forces, or electrostatic interactions. In parallel with this, the negative entropy suggests an enhancement in system order, which may be attributable to the ligand accommodating itself within the binding site on the protein and the limitation of water molecules. This thermodynamic profile suggests that a stable, well-matched complex with a specific binding character is formed.

**Figure 11:**
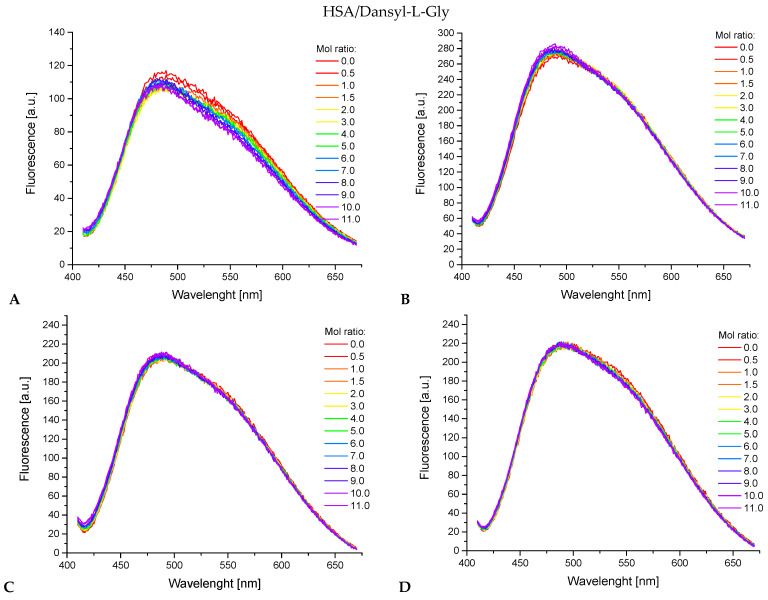
The fluorescence quenching of HSA/Dansyl-L-Gly after the addition of the respective compounds **3b**, **3c**, **3d**, and **3g** is shown in plots (**A**–**D**), respectively.

**Figure 12:**
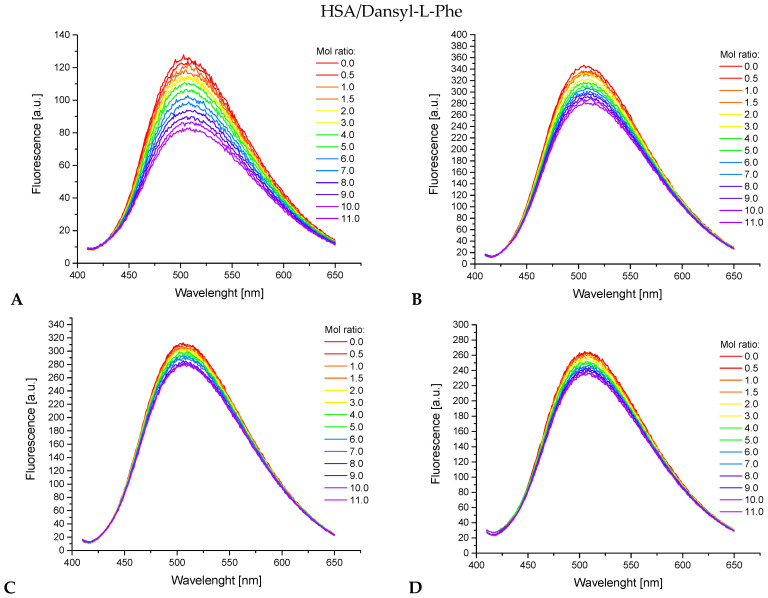
The fluorescence quenching of HSA/Dansyl-L-Phe after the addition of the respective compounds **3b**, **3c**, **3d**, and **3g** is shown in plots (**A**–**D)**, respectively.

The human serum albumin (HSA) has two main drug-binding sites, known as Sudlow site I and Sudlow site II, located in subdomains IIA and IIIA of the albumin structure, respectively. Sudlow I demonstrates a preference for large, frequently heterocyclic, acidic or neutral ligands, such as warfarin. Conversely, Sudlow II has been observed to bind smaller and more hydrophobic molecules, such as ibuprofen and diazepam [40]. In addition to these main sites, HSA has the capacity to bind ligands in other regions, including histidine residues, the *N*-terminus of the molecule, and metal binding sites. However, it is most common for drugs to bind to Sudlow I and Sudlow II. In order to study binding to a specific site in the protein, fluorescent markers, Dansyl-L-Gly and Dansyl-L-Phe, were used. The Dansyl-L-Gly interacts with Sudllow I, while the Dansyl-L-Phe prefers Sudllow II [41,42]. The tested compounds were added in portions to the solution that contained the HSA/marker complex, and the fluorescence spectrum was recorded (Figure 11 and Figure 12). The percentage of compound exchange in the albumin pocket was calculated using Equation (6):

(6) $$displacement\;\%\;=\frac{F^{0}\;-F\;}{F^{0}\;}$$

where *F*^0^ is the maximum fluorescence intensity of HSA/Dansyl-L-Gly complex, and *F* is the maximum fluorescence intensity after a quencher addition. The calculated data were collected in Table 6. It was observed that only compound **3b** displaces from the Dansyl-L-Gly binding site, as indicated by a decrease in fluorescence intensity with increasing concentration of this derivative, but only to approximately 8%. However, for compounds **3c**, **3d**, and **3g**, no quenching occurs at all. The band intensity increases slightly, which may be due to the dilution of the solution. The dilution of the sample leads to an increase in the average distance between molecules. This, in turn, results in a reduction in the number of collisions and reabsorptions, thereby enhancing the efficiency of fluorescence emission [38]. Nevertheless, the binding site for Dansyl-L-Phe was displaced by all of the tested derivatives. This finding indicates that the preferred binding site is Sudllow II for the series of pyridine-3-carboxamide derivatives under investigation.

### 2.6. Anticancer Activity

As mentioned earlier, MMP-13 is overexpressed and functionally significant in various cancers, including melanoma and colorectal carcinoma. For instance, high MMP-13 levels have been correlated with metastasis and poor survival in melanoma patients [6]. In colorectal cancer, elevated MMP-13 activity has been observed in tumors compared to normal mucosa, and its expression is associated with a worse prognosis [43]. Based on computational studies, four out of the eleven designed compounds showing the most promising predicted activity against MMP-13 were selected for biological evaluation. For the MTT assay, NHDF cells were used as the healthy reference cell line, while A375 (melanoma) and HT-29 (colorectal adenocarcinoma cancer) cell lines were chosen as cancer models.

**Figure 13:**
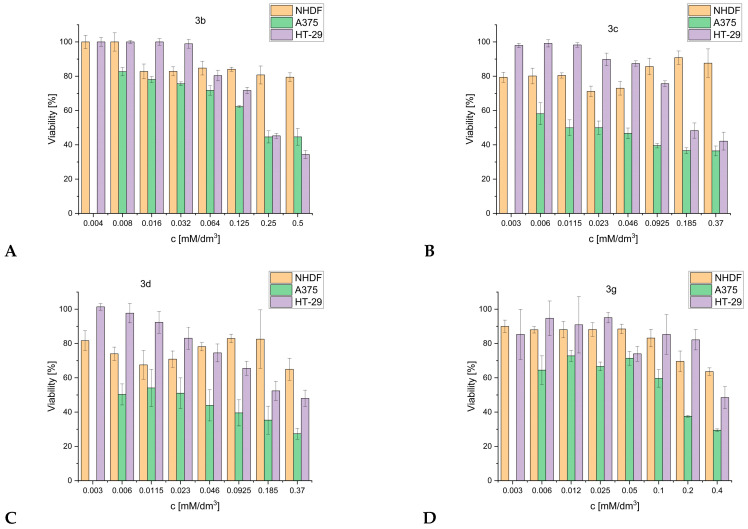
Cytotoxicity plots of designed compounds **3b** (**A**), **3c** (**B**), **3d** (**C**), and **3g** (**D**).

As shown in Figure 13, within the tested concentration range, none of the compounds reduced NHDF viability by 50% under the applied experimental conditions. All tested compounds maintained NHDF viability above 63% (observed for compound **3d**). Among them, compound **3b** deserves particular attention, as it exhibited the lowest toxicity toward healthy cells. At a concentration of 0.5 mM, the calculated selectivity ratios (A375|HT-29:NHDF) were 0.56 and 0.43, respectively, indicating the highest observed selectivity toward cancer cells.

In contrast, although compounds **3c** and **3d** caused the strongest reduction in cancer cell viability, they also displayed the highest toxicity toward healthy NHDF, resulting in the lowest selectivity indices. This effect may be attributed to the close structural similarity between these derivatives, resulting from the presence of an electron-withdrawing chlorine substituent in the aromatic ring. Compound **3g** demonstrated only moderate anticancer potential, with a less pronounced but still noticeable selectivity profile. Among the tested lines, melanoma (A375) cells appeared generally more sensitive to the compounds, which aligns with prior findings on MMP-13-driven invasiveness in melanoma.

## 3. Materials and Methods

### 3.1. Chemistry

#### 3.1.1. General Comments

All solvents and chemical reagents were purchased from commercial suppliers (Chemat, Gdańsk, Poland; Alchem, Wrocław, Poland) and were used without further purification. Dry solvents were received due to the standard procedures. Reaction progress was monitored by Thin Layer Chromatography (TLC) technique on silica gel coated aluminum sheets (Silica Gel 60 F_254_). Spots on the TLC plates were visualized by UV light at 254/366 nm. The melting points of final compounds were determined on the Electrothermal Mel-Temp 1101D apparatus (Cole-Parmer, Vernon Hills, IL, USA) using the open capillary method and were uncorrected. ^1^H NMR (600 MHz) and ^13^C NMR (151 MHz) spectra were recorded using Bruker 600 MHz NMR spectrometer (Bruker Analytische Messtechnik GmbH, Rheinstetten, Germany) in DMSO-*d*_6_, with tetramethylsilane (TMS) as an internal reference. Chemical shifts (*δ*) were reported in ppm, and multiplicities of NMR signals are designated as s (singlet), d (doublet), t (triplet), q (quartet), and m (multiplet, for unresolved lines). Coupling constants (*J*) were reported in hertz. To record and read spectra, the program TopSpin 4.1.4 (Bruker Daltonik, GmbH, Bremen, Germany) was used. FT-IR spectra were measured on Nicolet iS50 FT-IR Spectrometer (Thermo Fisher Scientific, Waltham, MA, USA). Frequencies were reported in cm^−1^. All samples were solid, and spectra were read by OMNIC Spectra 2.0 (Thermo Fisher Scientific, Waltham, MA, USA). Mass spectra (MS) were recorded using the Bruker Daltonics Compact ESI-Mass Spectrometer (Bruker Daltonik, GmbH, Bremen, Germany), operating in the positive ion mode. The analyzed compounds were dissolved in a methanol/chloroform mixture. Theoretical monoisotopic masses of ions were calculated (calcd.) using Bruker Compass Data Analysis 4.2 software (Bruker Daltonik GmbH, Bremen, Germany).

#### 3.1.2. General Procedure for the Synthesis of 4,6-Dimethyl-*N*-{2-[(substituted-methyl)amino]-2-oxoethyl}-2-sulfanylpyridine-3-carboxamide Derivatives **3a**–**k**

4,6-Dimethyl-*N*-[(5-sulfanylidene-4,5-dihydro-1,3,4-oxadiazol-2-yl)methyl]-2-sulfanyl-pyridine-3-carboxamide **2** (0.001 mol) and the appropriate amine derivative (0.001 mol) were refluxed in 20 mL of ethanol containing 36% formaldehyde (0.2 mL) for 2 h. After cooling to room temperature, the resulting precipitate was filtered off and allowed to dry. The crude product was isolated and, if necessary, purified by crystallization from ethanol.

**3a** 4,6-dimethyl-*N*-{2-[(4-trifluoromethylbenzyl)amino]-2-oxoethyl}-2-sulfanylpyridine-3-carboxamide.

Pale yellow solid (45%); m.p. 275-7 °C. **FT-IR** (selected lines, γ_max_, cm^−1^): 3188 (NH), 3043 (aromatic C-H stretching), 2932 (aliphatic C-H stretching), 1654, 1635, 1618 (C=O, C=N). **^1^H NMR** (600 MHz, DMSO-*d*_6_): δ = 2.11 (s, 3H, CH_3-pyridine_), 2.32 (s, 3H, CH_3-pyridine_), 3.84 (d, 2H, CH_2_, *J* = 6 Hz), 4.42 (d, 2H, CH_2_, *J* = 6 Hz), 6.63 (s, 1H, H_-pyridine_), 7.51 (d, 2H, Ar, *J* = 6 Hz), 7.66 (d, 2H, Ar, *J* = 6 Hz), 8.83 (t, 1H, NH, *J* = 6 Hz), 9.17 (t, 1H, NH, *J* = 6 Hz), 13.51 (s, 1H, SH). **^13^C NMR** (151 MHz, DMSO-*d*_6_): δ = 18.80, 19.61, 41.79, 42.92, 116.17, 124.00, 125.56, 128.38, 136.78, 144.61, 147.31, 149.06, 167.36, 169.74, 173.43. **HR-MS** calculated for C_18_H_18_F_3_N_3_O_2_S [M + H]^+^ 398.1145, found: 398.1156.

**3b** 4,6-dimethyl-*N*-{2-[(4-phenoxybenzyl)amino]-2-oxoethyl}-2-sulfanylpyridine-3-carboxamide.

White solid (30%); m.p. 205-8 °C. **FT-IR** (selected lines, γ_max_, cm^−1^): 3193 (NH), 3037 (aromatic C-H stretching), 2923 (aliphatic C-H stretching), 1667, 1653, 1618 (C=O, C=N). **^1^H NMR** (600 MHz, DMSO-*d*_6_): δ = 2.10 (s, 3H, CH_3-pyridine_), 2.32 (s, 3H, CH_3-pyridine_), 3.82 (d, 2H, CH_2_, *J* = 6 Hz), 4.32 (d, 2H, CH_2_, *J* = 6 Hz), 6.62 (s, 1H, H_-pyridine_), 6.81–7.40 (m, 8H, Ar), 8.79 (t, 1H, NH, *J* = 6 Hz), 9.07 (t, 1H, NH, *J* = 6 Hz), 13.50 (s, 1H, SH). **^13^C NMR** (151 MHz, DMSO-*d*_6_): δ = 18.85, 19.58, 41.91, 42.92, 116.14, 117.34, 118.30, 119.03, 122.98, 123.85, 130.27, 130.59, 136.91, 142.00, 147.28, 148.97, 156.95, 157.18, 167.27, 169.52, 173.64. **HR-MS** calculated for C_23_H_23_N_3_O_3_S [M + H]^+^ 422.1533, found: 422.1573.

**3c** *N*-{2-[(4-chlorobenzyl)amino]-2-oxoethyl}-4,6-dimethyl-2-sulfanylpyridine-3-carboxamide.

White solid (47%); m.p. 280-2 °C. **FT-IR** (selected lines, γ_max_, cm^−1^): 3200 (NH), 3043 (aromatic C-H stretching), 2926 (aliphatic C-H stretching), 1654, 1633, 1618 (C=O, C=N). **^1^H NMR** (600 MHz, DMSO-*d*_6_): δ = 2.10 (s, 3H, CH_3-pyridine_), 2.32 (s, 3H, CH_3-pyridine_), 3.82 (d, 2H, CH_2_, *J* = 6 Hz), 4.31 (d, 2H, CH_2_, *J* = 6 Hz), 6.62 (s, 1H, H_-pyridine_), 7.30 (d, 2H, Ar, *J* = 6 Hz), 7.35 (d, 2H, Ar, *J* = 12Hz), 8.81 (t, 1H, NH, *J* = 6 Hz), 9.09 (t, 1H, NH, *J* = 6 Hz), 13.50 (s, 1H, SH). **^13^C NMR** (151 MHz, DMSO-*d*_6_): δ = 18.80, 19.55, 41.64, 42.95, 116.26, 128.67, 129.79, 131.96, 136.75, 138.63, 147.37, 149.06, 167.39, 169.74, 173.59. **HR-MS** calculated for C_17_H_18_ClN_3_O_2_S [M + H]^+^ 364.0881, found: 364.0868.

**3d** *N*-{2-[(2-chlorobenzyl)amino]-2-oxoethyl}-4,6-dimethyl-2-sulfanylpyridine-3-carboxamide.

White solid (33%); m.p. 285-7 °C. **FT-IR** (selected lines, γ_max_, cm^−1^): 3187 (NH), 3047 (aromatic C-H stretching), 2856 (aliphatic C-H stretching), 1654, 1634, 1620 (C=O, C=N). **^1^H NMR** (600 MHz, DMSO-*d*_6_): δ = 2.11 (s, 3H, CH_3-pyridine_), 2.31 (s, 3H, CH_3-pyridine_), 3.86 (d, 2H, CH_2_, *J* = 6 Hz), 4.38 (d, 2H, CH_2_, *J* = 6 Hz), 6.62 (s, 1H, H_-pyridine_), 7.28–7.29 (m, 2H, Ar), 7.41–7.44 (m, 2H, Ar), 8.85 (t, 1H, NH, *J* = 6 Hz), 9.08 (t, 1H, NH, *J* = 6 Hz), 13.50 (s, 1H, SH). **^13^C NMR** (151 MHz, DMSO-*d*_6_): δ = 18.80, 19.45, 42.95, 116.17, 127.44, 129.04, 129.42, 132.24, 136.37, 136.84, 147.27, 149.06, 167.29, 169.74, 173.59. **HR-MS** calculated for C_17_H_18_ClN_3_O_2_S [M + H]^+^ 364.0881, found: 364.0837.

**3e** 4,6-dimethyl*-N*-{2-[(4-methoxybenzyl)amino]-2-oxoethyl}-2-sulfanylpyridine-3-carboxamide.

White solid (47%); m.p. 250-4 °C. **FT-IR** (selected lines, γ_max_, cm^−1^): 3210 (NH), 3041 (aromatic C-H stretching), 2923 (aliphatic C-H stretching), 1654, 1617 (C=O). **^1^H NMR** (600 MHz, DMSO-*d*_6_): δ = 2.10 (s, 3H, CH_3-pyridine_), 2.32 (s, 3H, CH_3-pyridine_), 3.72 (s, 3H, CH_3_), 3.80 (d, 2H, CH_2_, *J* = 6 Hz), 4.25 (d, 2H, CH_2_, *J* = 6 Hz), 6.61 (s, 1H, H_-pyridine_), 6.84 (d, 2H, Ar, *J* = 12Hz), 7.20 (d, 2H, Ar, *J* = 12Hz), 8.75 (t, 1H, NH, *J* = 6 Hz), 8.98 (t, 1H, NH, *J* = 6 Hz), 13.48 (s, 1H, SH). **^13^C NMR** (151 MHz, DMSO-*d*_6_): δ = 18.73, 19.61, 41.67, 42.86, 55.51, 114.10, 116.10, 129.26, 131.52, 136.91, 147.31, 148.87, 158.65, 167.36, 169.24, 173.62. **HR-MS** calculated for C_18_H_21_N_3_O_3_S [M + H]^+^ 360.1376, found: 360.1361.

**3f** 4,6-dimethyl*-N*-{2-[(2-methoxybenzyl)amino]-2-oxoethyl}-2-sulfanylpyridine-3-carboxamide.

White solid (38%); m.p. 242-4 °C. **FT-IR** (selected lines, γ_max_, cm^−1^): 3193 (NH), 3050 (aromatic C-H stretching), 2923 (aliphatic C-H stretching), 1654, 1617 (C=O). **^1^H NMR** (600 MHz, DMSO-*d*_6_): δ = 2.10 (s, 3H, CH_3-pyridine_), 2.31 (s, 3H, CH_3-pyridine_), 3.80 (s, 3H, CH_3_), 3.83 (d, 2H, CH_2_, *J* = 6 Hz), 4.27 (d, 2H, CH_2_, *J* = 6 Hz), 6.61 (s, 1H, H_-pyridine_), 6.86 (m, 1H, Ar), 6.94–6.96 (m, 1H, Ar), 7.23 (m, 2H, Ar), 8.80 (t, 1H, NH, *J* = 6 Hz), 8.86 (t, 1H, NH, *J* = 6 Hz), 13.48 (s, 1H, SH). **^13^C NMR** (151 MHz, DMSO-*d*_6_): δ = 18.75, 19.55, 37.28, 42.94, 55.73, 110.68, 116.01, 120.44, 126.93, 128.31, 128.46, 136.84, 147.18, 148.87, 156.96, 167.29, 169.49, 173.68. **HR-MS** calculated for C_18_H_21_N_3_O_3_S [M + H]^+^ 360.1376, found: 360.1367.

**3g** 4,6-dimethyl-*N*-[2-(benzylamino)-2-oxoethyl]-2-sulfanylpyridine-3-carboxamide.

White solid (48%); m.p. 269-71 °C. **FT-IR** (selected lines, γ_max_, cm^−1^): 3199 (NH), 3056 (aromatic C-H stretching), 2923 (aliphatic C-H stretching), 1655, 1619 (C=O). **^1^H NMR** (600 MHz, DMSO-*d*_6_): δ = 2.11 (s, 3H, CH_3-pyridine_), 2.32 (s, 3H, CH_3-pyridine_), 3.83 (d, 2H, CH_2_, *J* = 6 Hz), 4.32 (d, 2H, CH_2_, *J* = 6 Hz), 6.62 (s, 1H, H_-pyridine_), 7.22–7.30 (m, 5H, Ar), 8.79 (t, 1H, NH, *J* = 6 Hz), 9.05 (t, 1H, NH, *J* = 6 Hz), 13.50 (s, 1H, SH). **^13^C NMR** (151 MHz, DMSO-*d*_6_): δ = 18.80, 19.53, 42.18, 42.83, 116.10, 127.10, 127.84, 128.66, 136.82, 139.57, 147.28, 149.02, 167.45, 169.47, 173.69. **HR-MS** calculated for C_17_H_19_N_3_O_2_S [M + H]^+^ 330.1271, found: 330.1266.

**3h** 4,6-dimethyl-*N*-{2-[(4-trifluoromethoxybenzyl)amino]-2-oxoethyl)-2-sulfanylpyridine-3-carboxamide.

White solid (53%); m.p. 284-6 °C. **FT-IR** (selected lines, γ_max_, cm^−1^): 3191 (NH), 3048 (aromatic C-H stretching), 2974 (aliphatic C-H stretching), 1653, 1621 (C=O). **^1^H NMR** (600 MHz, DMSO-*d*_6_): δ = 2.11 (s, 3H, CH_3-pyridine_), 2.32 (s, 3H, CH_3-pyridine_), 3.83 (d, 2H, CH_2_, *J* = 6 Hz), 4.35 (d, 2H, CH_2_, *J* = 6 Hz), 6.63 (s, 1H, H_-pyridine_), 7.29 (d, 2H, Ar, *J* = 6 Hz), 7.41 (d, 2H, Ar, *J* = 6 Hz), 8.82 (t, 1H, NH, *J* = 6 Hz), 9.13 (t, 1H, NH, *J* = 6 Hz), 13.51 (s, 1H, SH). **^13^C NMR** (151 MHz, DMSO-*d*_6_): δ = 18.80, 19.53, 41.54, 42.92, 116.28, 121.32, 129.58, 136.91, 139.21, 147.28, 147.64, 149.11, 167.45, 169.65, 173.50. **HR-MS** calculated for C_18_H_18_F_3_N_3_O_3_S [M + H]^+^ 414.1094, found: 414.1081.

**3i** 4,6-dimethyl*-N*-(2-{[(furan-2-yl)methyl]amino}-2-oxoethyl)-2-sulfanylpyridine-3-carboxamide.

White solid (47%); m.p. 282-5 °C. **FT-IR** (selected lines, γ_max_, cm^−1^): 3183 (NH), 3054 (aromatic C-H stretching), 2970 (aliphatic C-H stretching), 1652, 1617 (C=O). **^1^H NMR** (600 MHz, DMSO-*d*_6_): δ = 2.10 (s, 3H, CH_3-pyridine_), 2.31 (s, 3H, CH_3-pyridine_), 3.80 (d, 2H, CH_2_, *J* = 6 Hz), 4.30 (d, 2H, CH_2_, *J* = 6 Hz), 6.25 (s, 1H, H_-furan_), 6.37 (s, 1H, H_-furan_), 6.60 (s, 1H, H_-pyridine_), 7.54 (s, 1H, H_-furan_), 8.74 (t, 1H, NH, *J* = 6 Hz), 8.91 (t, 1H, NH, *J* = 6 Hz), 13.48 (s, 1H, SH). **^13^C NMR** (151 MHz, DMSO-*d*_6_): δ = 18.83, 19.55, 35.67, 42.75, 107.60, 110.93, 115.97, 136.90, 142.61, 147.15, 148.86, 152.46, 167.30, 169.35, 173.67. **HR-MS** calculated for C_15_H_17_N_3_O_3_S [M + H]^+^ 320.1063, found: 320.1064.

**3j** 4,6-dimethyl-*N*-(2-{[(oxolan-2-yl)methyl]amino}-2-oxoethyl)-2-sulfanylpyridine-3-carboxamide.

Pale yellow solid (53%); m.p. 260-1 °C. **FT-IR** (selected lines, γ_max_, cm^−1^): 3210 (NH), 2973, 2936 (aliphatic C-H stretching), 1654, 1626 (C=O, C=N). **^1^H NMR** (600 MHz, DMSO-*d*_6_): δ = 1.56–1.59 (m, 1H, oxolane), 1.77–1.88 (m, 3H, oxolane), 2.10 (s, 3H, CH_3-pyridine_), 2.32 (s, 3H, CH_3-pyridine_), 3.10–3.12 (m, 1H, CH_2_), 3.21–3.23 (m, 1H, CH_2_), 3.58–3.59 (m, 1H, oxolane), 3.76–3.77 (m, 1H + 2H, oxolane + CH_2_), 3.84–3.86 (m, 1H, oxolane), 6.60 (s, 1H, H_-pyridine_), 8.46 (t, 1H, NH, *J* = 6 Hz), 8.70 (t, 1H, NH, *J* = 6 Hz), 13.47 (s, 1H, SH). **^13^C NMR** (151 MHz, DMSO-*d*_6_): δ = 18.76, 19.61, 25.47, 29.19, 42.79, 42.90, 67.61, 77.40, 115.96, 136.95, 147.23, 149.11, 167.39, 169.74, 173.96. **HR-MS** calculated for C_15_H_21_N_3_O_3_S [M + H]^+^ 324.1376, found: 324.1375.

**3k** 4,6-dimethyl*-N*-{2-[(4-trifluoromethylsulfanylbenzyl)amino]-2-oxoethyl}-2-sulfanylpyridine-3-carboxamide.

White solid (47%); m.p. 295-8 °C. **FT-IR** (selected lines, γ_max_, cm^−1^): 3186 (NH), 3047 (aromatic C-H stretching), 2978 (aliphatic C-H stretching), 1654, 1621 (C=O). **^1^H NMR** (600 MHz, DMSO-*d*_6_): δ = 2.12 (s, 3H, CH_3-pyridine_), 2.33 (s, 3H, CH_3-pyridine_), 3.84 (d, 2H, CH_2_, *J* = 6 Hz), 4.40 (d, 2H, CH_2_, *J* = 6 Hz), 6.63 (s, 1H, H_-pyridine_), 7.45 (d, 2H, Ar, *J* = 6 Hz), 7.65 (d, 2H, Ar, *J* = 6 Hz), 8.85 (t, 1H, NH, *J* = 6 Hz), 9.17 (t, 1H, NH, *J* = 6 Hz), 13.52 (s, 1H, SH). **^13^C NMR** (151 MHz, DMSO-*d*_6_): δ = 18.77, 19.54, 41.64, 42.90, 116.16, 121.36, 129.13, 136.61, 136.79, 143.73, 147.30, 149.04, 167.40, 169.72, 173.40. **HR-MS** calculated for C_18_H_18_F_3_N_3_O_2_S_2_ [M + H]^+^ 430.0865, found: 430.0870.

### 3.2. Computational Studies

#### 3.2.1. Geometry and Properties of Designed Compounds

The structures of novel compounds were built in Avogadro v. 1.2.0 program [44]. Next, input files for geometry optimization were prepared. The calculations were performed in Gaussian 16.C.02. at the B3LYP/6-31++G** level of theory using PCM solvent model [45,46].

The energy of the frontier molecular orbitals was calculated at the same level of theory, and global reactivity descriptors such as electron affinity (EA) and ionization potential (IP) were estimated. The ionization potential is defined as the energy required to remove an electron from the molecule (-E_HOMO_), while the electron affinity is defined as the energy released when a molecule gains an electron (-E_LUMO_). Next parameter, molecular hardness (**η**) can be calculated using expression (7), resulting from Janak’s theorem [47]:

(7) $$\eta \;=\;\frac{IP-EA}{2}$$

The corresponding descriptor, molecular softness (**s**), is obtained using Equation (8).

(8) $$s=\frac{1}{2\eta }$$

An additional key parameter is the global electrophilicity index (ω), which can be expressed by the following Equation (9) [48].

(9) $$\omega =\frac{\mu^{2}}{2\eta }$$

Chemical potential (*μ*) and electronegativity (χ) are also important parameters derived from orbital energies. The chemical potential is associated with charge transfer in the ground state and can be expressed as shown in Equation (10).

(10) $$\mu =\frac{-\left(IP+EA\right)}{2}$$

The electronegativity index reflects the tendency of an atom to attract electrons in a covalent bond and can be quantified using Equation (11).

(11) $$\chi =\frac{\left(IP+EA\right)}{2}$$

#### 3.2.2. Molecular Docking

The protein structures were obtained from the Protein Data Bank [49]. For further analysis MMP-13 (PDB ID: 5BPA) and MMP-8 (PDB ID: 1BZS) structures were used [30,50]. Due to the presence of a catalytic zinc ion in both enzymes, docking was performed using AutoDock Tools 1.5.7 with the AutoDockZN force field, which enables more accurate treatment of zinc–ligand coordination [51,52].

**Figure 14:**
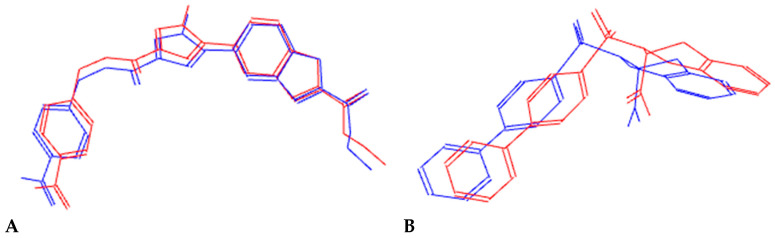
Illustration of results from re-docking. Co-crystalized ligand in blue and re-docked pose as red for 4FU (**A**) and BSI (**B**), respectively.

During docking to MMP-13, we used a protocol involving a grid box with dimensions of 118 × 80 × 80 Å and a spacing of 0.2 Å. The grid box was centered at x = −12.311, y= 22.963, and z = 47.314. The procedure was performed using a genetic algorithm with default parameters and 100 runs of the Lamarcikan GA. Additionally, a novel partial charge of +1200 eV was assigned to the zinc atom (ZN) to improve the accuracy of zinc-ligand interactions [53]. The same docking protocol was applied to MMP-8, with the grid box centered at x = 27.860, y = 55.950, and z = 47.340. To verify the accuracy of the docking procedure, we performed a re-docking of the co-crystalized ligand and compared the predicted pose with the crystal of both proteins. The RMSD values were calculated using the LigRMSD 1.0software [54]. Their values were 0.63 Å for MMP-13 and 1.49 Å for MMP-8, indicating good reproduction of the experimental binding poses. (See Figure 14) The binding modes of designed compounds were visualized using Discovery Studio visualizer 2021 (Dassault Systèmes Corporate, Dassault Systèmas, Waltham, MA, USA, https://discover.3ds.com/ accessed on 1 October 2025).

#### 3.2.3. Molecular Dynamic Simulations

To perform 100 ns of molecular dynamics simulations (MD) for MMP-13/**3a**–**k** and MMP-8/**3a**–**k** complexes, we used GROMACS version 2021.2-MODIFIED [55]. Systems were built using the web-based CHARMM-GUI platform (http://www.charmm-gui.org/) [56]. Ligand poses with the best binding affinity obtained from molecular docking were used as starting points for simulations. Topology files were generated using CHARMM General Force Field (CGenFF) version 2.5.1 [57]. The local environment was set up at 7 pH. Accordingly, the protonation states of ionizable (acidic and basic) residues were assigned to reflect physiological conditions. All molecules were solvated in a water box (rectangular) using the TIP3P water model [58] with an edge distance of 10 Å. To neutralize the system’s net charge sodium and chloride ions were added at a concentration of 0.15 M. Ions were inserted into regions of optimal electrostatic potential by replacing water molecules. In the next step, energy minimization was conducted using the steepest descent algorithm with a tolerance of 1000 kJ/mol*nm. After convergence of the system, simulations were performed using the NVT ensemble for 12,500 steps, and NPT ensemble simulations were conducted under periodic boundary conditions. The CHARMM36m force field was applied, and for temperature and pressure, we used Berendsen thermostat and barostat (303.15 K/1 bar) [59,60].

To calculate binding free energy, the GMX_MMPBSA tool was used [61]. All plots were made using Origin Pro 2024 v.10.1 OriginLab Corporation, Northampton, MA, USA [62].

#### 3.2.4. ADMET Evaluation

For the prediction of ADMET parameters (absorption, distribution, metabolism, excretion, and toxicity), we used the online tool ADMETlab 3.0, available at [https://admetlab3.scbdd.com/] [63].

### 3.3. MMP-13 and MMP-8 Inhibitory Assay

The inhibitory activity of the tested compounds was evaluated using the MMP-13 inhibitor screening assay kit (fluorometric) (Abcam, ab139451). The assay performed according to the manufacturer’s instructions. The designed compounds were tested at concentrations ranging from 1.8 to 4.6 µM, to determine their inhibitory activity. Additionally, the MMP-8 inhibitory screening assay kit (fluorometric) (Abcam, ab139453) was used to assess the inhibitory affinity of the designed compound toward MMP-8.

### 3.4. Fluorescence and UV-Vis Studies

#### 3.4.1. Binding to Human Serum Albumin

To perform the UV-Vis and fluorometric titration measurements, all compounds were dissolved in dimethyl sulfoxide (DMSO) at a concentration of 1⸱10^−3^ mol/dm^3^. A 0.01 mol/dm^3^ phosphate-buffered saline (PBS) solution with a pH of 7.4 (Sigma-Aldrich Chemie GmbH, St. Louis, MO, USA) was used to prepare a 1 × 10^−6^ mol/dm^3^ solution of human serum albumin (HSA) (Sigma-Aldrich Chemie GmbH, St. Louis, MO, USA). The fluorescence study was performed on a Cary Eclipse 500 spectrophotometer (Agilent, Santa Clara, CA, USA). The fluorometric spectra were recorded in a quartz cuvette with a 10 mm path length over an emission range of 280–400 nm, with an excitation wavelength of 280 nm and a PMT voltage of 560 V. The UV-Vis spectra were recorded on a Jasco V-750 spectrophotometer (Jasco, Tokyo, Japan) with the following parameters: a wavelength range of 190–400 nm, a data interval of 0.1 nm, a scan speed of 400 nm/min, and automatic baseline correction. Quartz cuvettes with an optical path length of 10 mm were used. All measurements were carried out at three temperatures: 298, 303, and 308 K. The titrations of the protein solution with the studied compounds were performed in 0.5 molar increments, from 0.0 to 2.0 followed by 1.0 increments up to a final molar ratio of 11.0.

#### 3.4.2. Determination of Specific Pocket in Human Serum Albumin

Solutions of Dansyl-L-Phe (Chem-Norm Biotech, Wuhan, China) and Dansyl-L-Gly (Sigma-Aldrich, Tokyo, Japan) were prepared by dissolving them separately in ethanol to a final concentration of 1·10^−3^ mol/dm^3^. The HSA solution was prepared in the same way as for titration binding measurements. Spectra were recorded at room temperature using a Cary Eclipse 500 spectrophotometer (Agilent, Santa Clara, CA, USA) with an excitation wavelength of 350 nm, and an emission range of 410–670 nm and the PMT voltages were (V) 700 and 650 for Dansyl-L-Gly and Dansyl-L-Phe, respectively. The complexes were prepared by mixing the HSA solution with the dansylated markers, and then adding the solution of the studied compounds from a ratio of 0 to 11.0 molar at the same increments, using the same protocol as in the spectroscopy studies.

### 3.5. Cell Culture

#### 3.5.1. Cell Lines Conditions

Experiments were conducted using two cancer cell lines and, as a control, a normal human dermal fibroblasts (NHDF; ATCC PCS-201-012). The A375 cell line (human melanoma, ATCC CRL-1619), and HT-29 cell line (human colorectal adenocarcinoma, ATCC HTB-38) were cultured in DMEM (Dulbecco’s Modified Eagle Medium, Institute of Immunology and Experimental Therapy (IITD), Wrocław, Poland) supplemented with 1% antibiotics (10 U/mL penicillin and 10 µg/mL streptomycin, Sigma-Aldrich, St Louis, MO, USA), 1% glutamine (Sigma-Aldrich, St Louis, MO, USA) and 10% fetal bovine serum (FBS, Gibco, Grand Island, NY, USA). For NHDF cultures, α-MEM (α-Minimum Essentials Medium, Institute of Immunology and Experimental Therapy (IITD), Poland) was used with the same supplement combination as for the cancer cell lines. All cells were maintained in a humidified incubator (INCO 105, Memmert GmbH, Schwabach, Germany) at 37 °C with 5% CO_2_ and 95% humidity. Cells were passaged routinely by trypsinization (twice a week). For the MTT (3-(4,5-simethylthiazol-2,5-diphenyltetrazolinum bromide), Sigma, St. Louis, MO, USA) assay, cells were used after 2–4 passages, when they reached approximately 80% confluence.

All tested compounds were dissolved in DMSO to obtain stock solutions with a final concentration of 50 mM (**3a**, **3b**, **3e**), 40 mM (**3f**, **3g**, **3i**, **3j**), and 35 mM (**3h**, **3k**). Due to low solubility, compounds **3c** and **3d** were dissolved in DMSO to a final concentration of 30 mM. In the next step, 10 µL of each stock solution was added to 990 µL of supplemented medium.

#### 3.5.2. MTT Assay

The MTT assay was performed in 96-well plates, with a seeding density of 1 × 10^4^ cells per well. All cell lines were incubated for 24 h in their respective media: α-MEM for (NHDF cells) and DMEM for (A375 and HT-29) cells. After incubation, the medium was removed and replaced with fresh medium containing the tested compounds at concentrations ranging from 0.004 mM to 0.5 mM. Control wells contained complete medium supplemented with 1% DMSO. Following 24 h of incubation at 37 °C, the medium was removed, and a standard MTT assay was carried out. Absorbance was measured spectrophotometrically at a wavelength of 570 nm using a BioTek SYNERGY H1 microplate reader (Agilent, Santa Clara, CA, USA).

## 4. Conclusions

Our studies provide a comprehensive overview of the design, synthesis, and biological evaluation of a new class of dimethylpyridine-3-carboxyamide derivatives that act as selective non-zinc chelating inhibitors of MMP-13. The compounds were successfully synthesized and characterized by detailed spectral analyses. Fluorescence spectroscopy studies demonstrated the ability of the compounds to bind human serum albumin, suggesting favorable plasma transport properties and systemic bioavailability. The predicted inhibitory activity of compounds was subsequently confirmed through enzymatic assays. Detailed structural analysis revealed that the selectivity of the designed ligands arises from a combination of complementary interactions within the MMP-13 selectivity pocket. The aliphatic chain of the compounds forms stabilizing hydrogen bonds with Thr245 and Thr247, while aromatic and heterocyclic moieties interact with Phe217, Leu218, and His222, anchoring ligands within the catalytic cleft. This structural complementarity results in the formation of π-stacking and hydrogen-bonding interactions that stabilize the complex and ensure strong and specific binding to MMP-13. In contrast, the narrower and less hydrophobic pocket of MMP-8 cannot accommodate the ligand, which explains the significantly reduced affinity and confirms the rational origin of selectivity. In parallel, the dimethylpyridine-3-carboxyamide derivatives exhibited cytotoxic activity against melanoma and colorectal cancer cell lines, indicating their potential anticancer properties. Such an effect may, at least in part, be related to the inhibition of MMP-13. It should be underlined that the precise molecular mechanism remains to be fully elucidated. Additional studies are planned to investigate this relationship in detail and determine whether MMP-13 modulation contributes directly to the observed anticancer activity. Further effort will also concentrate on optimizing the physicochemical and pharmacokinetic properties of the analyzed compounds. Conclusively, these findings provide a robust foundation for the ongoing development of next-generation MMP-13 inhibitors, which have potential applications in oncology and inflammatory disorders.

## Figures and Tables

**Table 1:** Reactivity parameters of designed compounds. Orbital energy values are expressed in electronvolts (eV).

Compound	E_HOMO_	E_LUMO_	GAP (_HOMO-LUMO_)	A	I	η	s	µ	χ	ω
**3a**	−6.53	−1.42	−5.11	1.42	6.53	2.56	0.39	−3.97	3.97	7.89
**3b**	−6.14	−1.20	−4.94	1.20	6.14	2.47	0.40	−3.67	3.67	6.73
**3c**	−6.21	−1.26	−4.95	1.26	6.21	2.47	0.40	−3.73	3.73	6.97
**3d**	−6.26	−1.16	−5.09	1.16	6.26	2.55	0.39	−3.71	3.71	6.88
**3e**	−6.12	−1.17	−4.95	1.17	6.12	2.47	0.40	−3.65	3.65	6.64
**3f**	−6.11	−1.59	−4.52	1.60	6.11	2.26	0.44	−3.85	3.85	7.42
**3g**	−6.15	−1.21	−4.95	1.21	6.15	2.47	0.40	−3.68	3.68	6.77
**3h**	−6.33	−1.21	−5.11	1.21	6.33	2.56	0.39	−3.77	3.77	7.10
**3i**	−6.26	−1.09	−5.17	1.09	6.26	2.58	0.39	−3.68	3.68	6.76
**3j**	−6.68	−1.64	−5.04	1.64	6.68	2.52	0.40	−4.16	4.16	8.65
**3k**	−6.35	−1.71	−4.64	1.71	6.35	2.32	0.43	−4.03	4.03	8.13

**Table 2:** ADMET properties of selected compounds.

Properties:	3b	3c	3d	3g
Lipinski Rule	Accepted	Accepted	Accepted	Accepted
TPSA	80.32	71.09	71.09	71.09
Absorption				
Caco-2 Permeability	−5.30	−5.10	−5.58	−5.30
MDCK Premeability	−4.65	−4.76	−4.80	−4.75
HIA	>30%	>30%	>30%	>30%
Distribution				
PPB	98.70%	98.30%	98.50%	97.70%
VD_ss_ [L/kg]	1.19	1.14	1.18	1.13
Excretion				
CL_plasma_ [mL/min/kg]	4.18	4.72	5.07	4.64
T_1/2_ [h]	0.52	0.47	0.48	0.47
Toxicity The output value is the probability of being toxic within the range of 0 to 1.				
hERG Blockers	0.11	0.04	0.03	0.04
AMES Toxicity	0.83	0.66	0.62	0.69
Rat Oral Acute Toxicity	0.25	0.32	0.40	0.34
Carcinogenicity	0.49	0.14	0.18	0.11

**Table 3:** The essential intermolecular interaction in the binding pocket of MMP-13 and MMP-8.

Enzyme	Hydrogen Bonds	π-Type Interactions with the Heterocyclic or Aromatic Ring of the Inhibitor
MMP-13	Lys140 Thr245 Thr247 Met253	Phe217 Leu218 His222
MMP-8	Leu160 Ala161	Tyr219 His197

**Table 4:** Binding free energy [kcal/mol]. Calculations performed using 1001 complex frames for each.

Compound:	MMP-13	MMP-8
**4FU**	−38.97	-
**3b**	−38.62	−19.48
**3c**	−36.61	−14.70
**3d**	−33.89	−19.35
**3g**	−21.64	−15.64

**Table 5:** The quenching, binding, and thermodynamic parameters of the interaction between HSA and compounds **3b**, **3c**, **3d** and **3g**.

Compound	Quenching			Binding			Thermodynamic		
	*T*	*K_SV_*·10^4^	*k_q_*·10^12^	log*K_b_*	*K_b_*·10^3^	n	Δ*G*^0^	Δ*H*^0^	Δ*S*^0^
	[K]	[dm^3^·mol^−1^]	[dm^3^·mol^−1^·s^−1^]		[dm^3^·mol^−1^]		[kJ·mol^−1^]	[kJ·mol^−1^]	[J·mol^−1^·K^−1^]
**3b**	297	1.21	1.21	3.52	3.32	0.89	−13.29	−402.16	−1282.70
	303	1.06	1.06	2.14	0.14	0.60			
	308	0.86	0.86	1.24	0.17	0.44			
**3c**	297	2.69	2.69	3.67	4.65	0.84	−18.07	−191.19	−571.09
	303	2.34	2.34	3.11	1.28	0.74			
	308	2.07	2.07	2.58	0.38	0.65			
**3d**	297	2.51	2.51	3.53	34.18	0.82	−17.39	−190.25	−570.21
	303	2.13	2.13	3.03	10.59	0.73			
	308	1.88	1.88	2.45	0.28	0.63			
**3g**	297	0.87	0.87	2.01	0.10	0.60	−8.86	−184.42	−579.14
	303	0.72	0.72	1.65	0.04	0.54			
	308	0.63	0.63	0.95	0.01	0.42			

**Table 6:** The percentage of markers Dansyl-L-Gly and Dansyl-L-Phe, displacement in the HSA binding pocket induced by the studied compounds **3b**, **3c**, **3d**, and **3g**.

Compound	Marker Displacement Percentage	
	HSA/Dansyl-L-Gly	HSA/Dansyl-L-Gly
**3b**	8.6%	35.2%
**3c**	-	18.5%
**3d**	-	10.6%
**3g**	-	11.1%

## Data Availability

The original contributions presented in this study are included in the article/Supplementary Material. Further inquiries can be directed to the corresponding authors.

## Supplementary Materials

The following supporting information can be downloaded at: https://www.mdpi.com/article/10.3390/molecules30244662/s1, Table S1. Visualizations of Nuclear Magnetic Resonance (NMR) spectra of compounds **3a**–**k** (DMSO-*d*_6_); Table S2. Visualizations of Fourier-Transform Infrared (FT-IR) spectra of compounds **3a**–**k**; Table S3. Visualizations of High Resolution Mass Spectrometry (HRMS) spectra of compounds **3a**-**k**; Table S4. HOMO and LUMO molecular orbitals of compounds **3a**–**k**; Table S5. 2D Interaction diagrams between the designed compounds and MMP-13 and MMP-8; Table S6. ADMET properties of compounds 3**a**–**k**; Figure S1. The Stearn-Volmer plots Double logarithm regression plot of the fluorescence quenching of HSA by compounds: A—**3b**, B—**3c**, C—**3d**, and D—**3g**; Figure S2. Double logarithm regression plot of the fluorescence quenching of HSA by compounds: A—**3b**, B—**3c**, C—**3d**, and D—**3g**.
