# Supplementary material for: New Dimethylpyridine-3-Carboxamide Derivatives as MMP-13 Inhibitors with Anticancer Activity
Source: Molecules. 2025 Dec 5;30(24):4662. doi: 10.3390/molecules30244662 (PMC12736008; doi:10.3390/molecules30244662)
Supplement: Supplementary file 1 [file molecules-30-04662-s001.zip › molecules-3994684-supplementary.docx]

**SUPPLEMENTARY MATERIAL**

New dimethylpyridine-3-carboxamide derivatives as MMP-13 inhibitors with anticancer activity

Remigiusz Płaczek ^1^*, Tomasz Janek ^2^ , Małgorzata Strzelecka ^3^, Aleksandra Kotynia ^1^, Piotr Świątek ^3^ and Żaneta Czyżnikowska ^1^*

^1^ Department of Basic Chemical Sciences, Faculty of Pharmacy, Wroclaw Medical University, Borowska 211, 50-556 Wroclaw, Poland; aleksandra.kotynia@umw.edu.pl

^2^ Department of Biotechnology and Food Microbiology, Faculty of Biotechnology and Food Science, Wroclaw University of Environmental and Life Sciences, Chełmońskiego 37, 51-630 Wroclaw, Poland

^3^ Department of Medicinal Chemistry, Faculty of Pharmacy, Wroclaw Medical University, Borowska 211, 50-556 Wroclaw, Poland; malgorzata.strzelecka@umw.edu.pl (M.S.); piotr.swiatek@umw.edu.pl (P.´S.)

***** Correspondence: remigiusz.placzek@student.umw.edu.pl (R.P.); zaneta.czyznikowska@umw.edu.pl (Ż.C.)

Table of contents:

**Table S1.** Visualizations of Nuclear Magnetic Resonance (NMR) spectra of compounds **3a**-**k** (DMSO‐d*6*) ................................................................................................................................ 2

**Table S2.** Visualizations of Fourier-Transform Infrared (FT-IR) spectra of compounds **3a**-**k**..13

**Table S3.** Visualizations of High Resolution Mass Spectrometry (HRMS) spectra of compounds **3a**-**k**……………………………………………………………………………... 17

**Table S4**. HOMO and LUMO molecular orbitals of compounds **3a**-**k** ................................... 23

**Table S5**. 2D Interaction diagrams between the designed compounds and MMP-13 and
MMP-8 ..................................................................................................................................... 26

**Figure S1**. The Stearn-Volmer plots Double logarithm regression plot of the fluorescence quenching of HSA by compounds: A - **3b**, B - **3c**, C - **3d**, and D - **3g** …………………,,….. 30

**Figure S2**. Double logarithm regression plot of the fluorescence quenching of HSA by compounds: A - **3b**, B - **3c**, C - **3d**, and D - **3g** ………………………………………,,…….. 31

**Table S6.** ADMET properties of compounds 3a-3k..................................................................32

**Table S1.** Visualizations of Nuclear Magnetic Resonance (NMR) spectra of compounds **3a**-**k** (DMSO‐d*6*).

| Comp. | ^1^H and ^1^C NMR spectra |
| --- | --- |
| **3a** | ^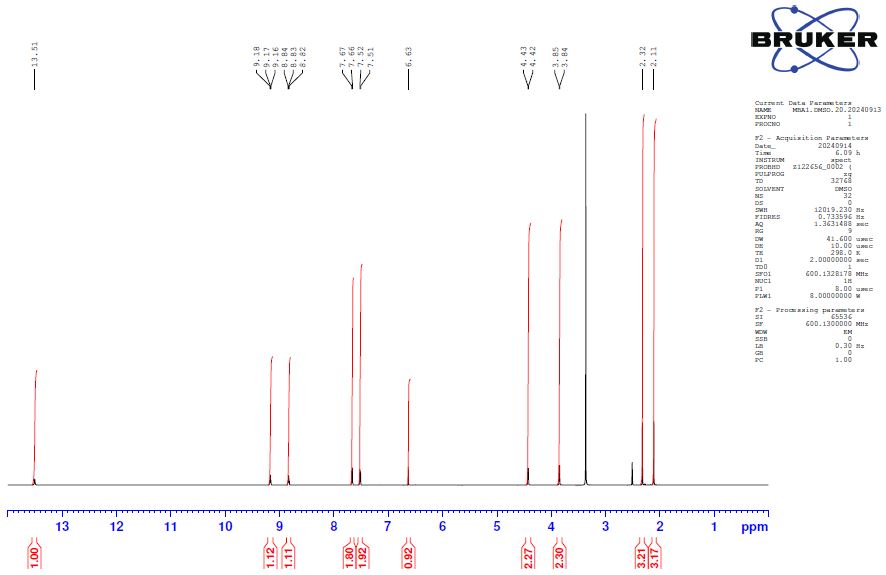^  ^1^H NMR of **3a**  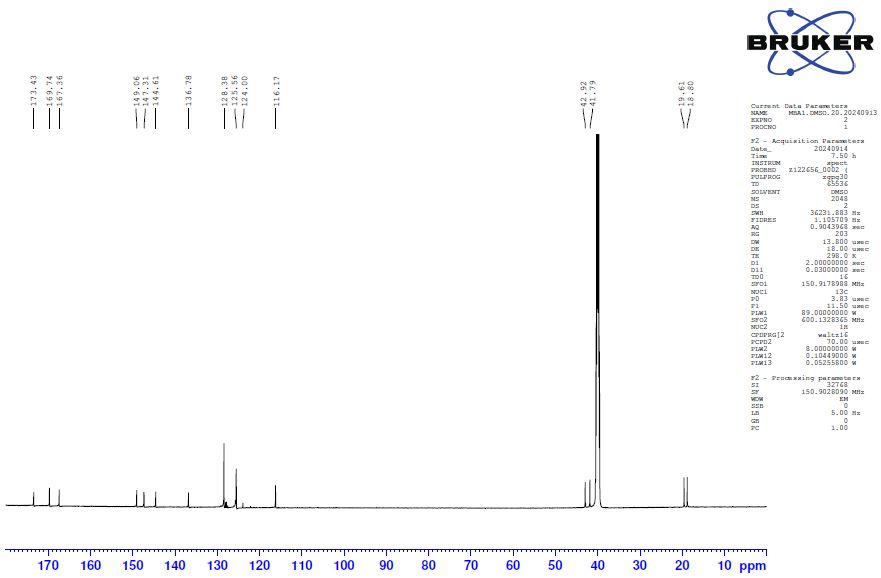  ^1^C NMR of **3a** |
| **3b** | 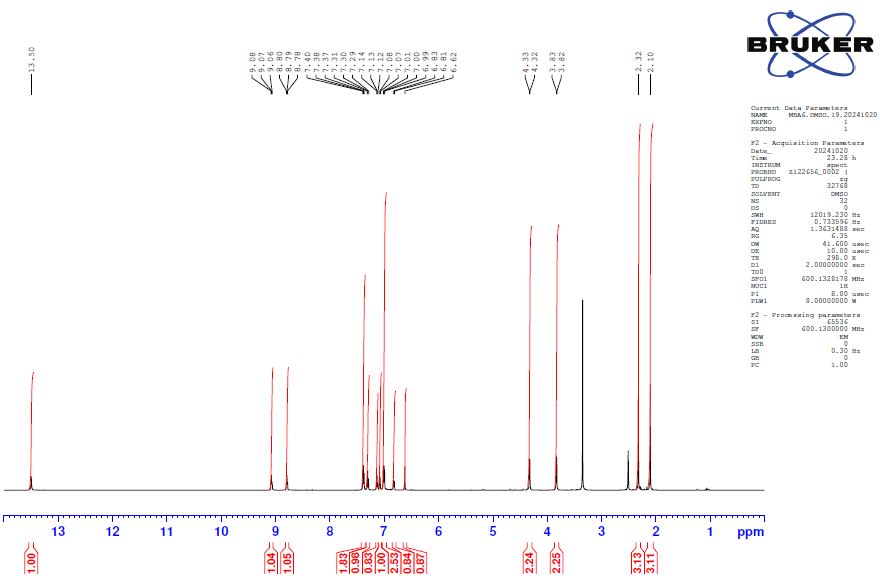  ^1^H NMR of **3b**  ^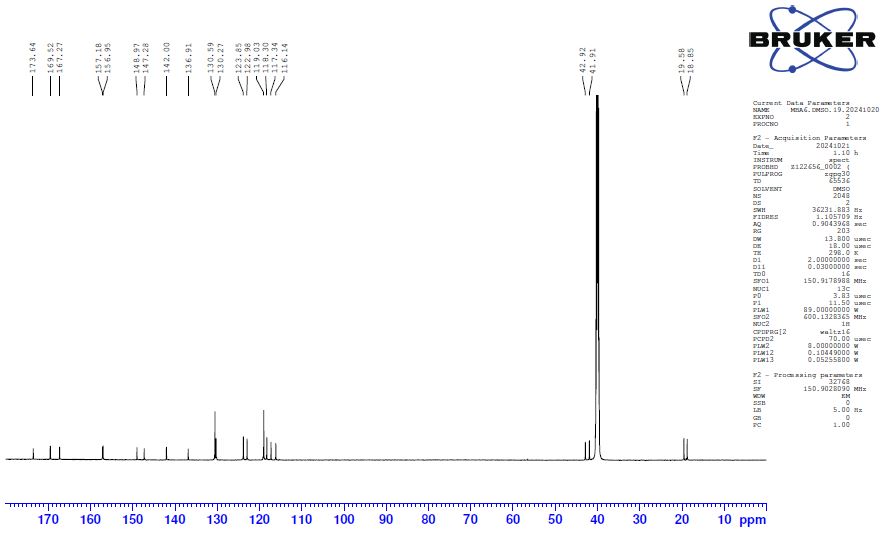^  ^1^C NMR of **3b** |
| **3c** | ^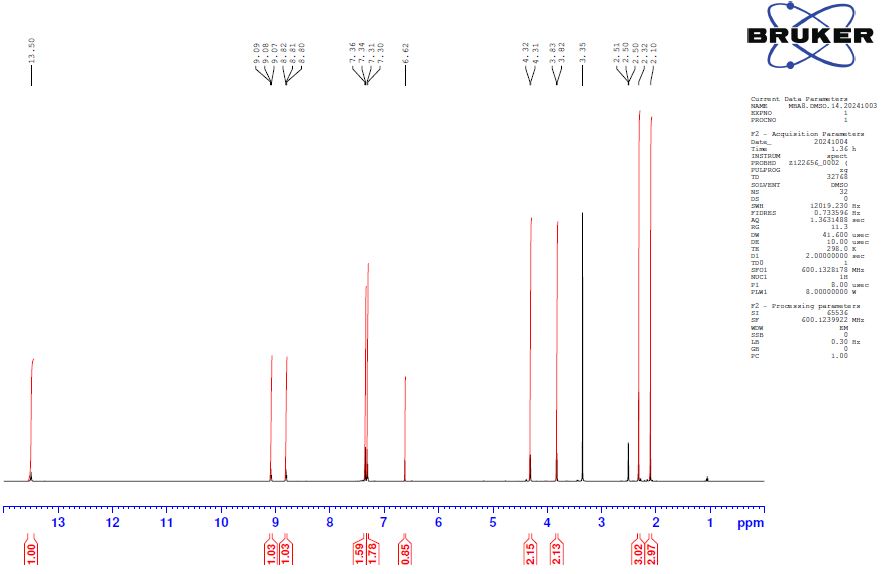^  ^1^H NMR of **3c**    ^1^C NMR of **3c** |
| **3d** | 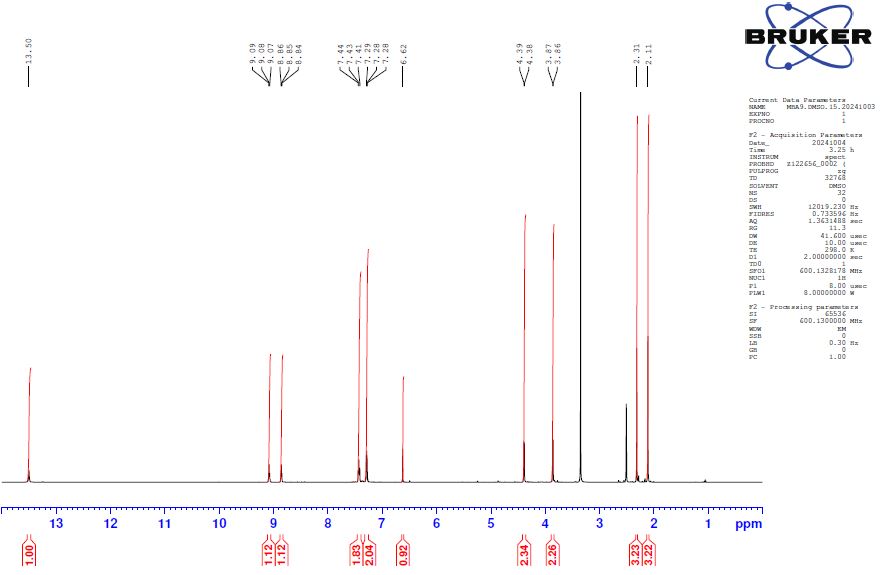  ^1^H NMR of **3d**  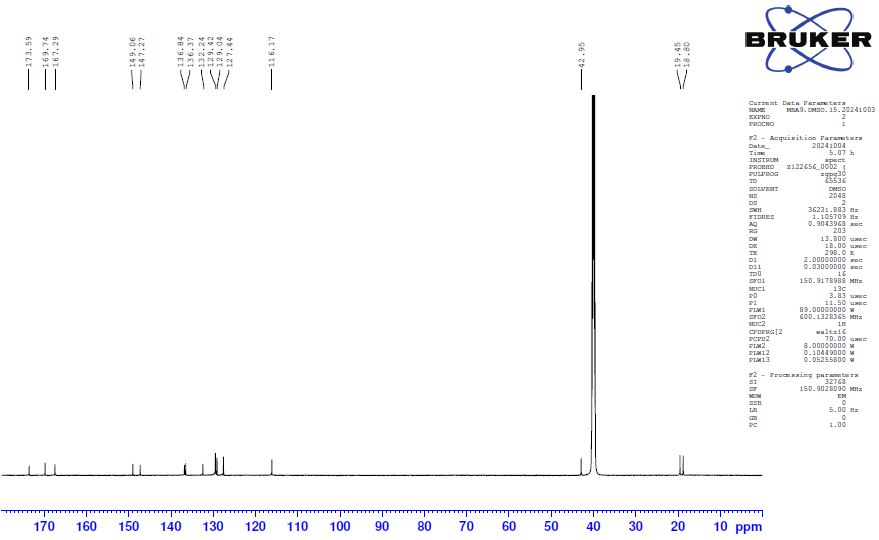  ^1^C NMR for **3d** |
| **3e** | ^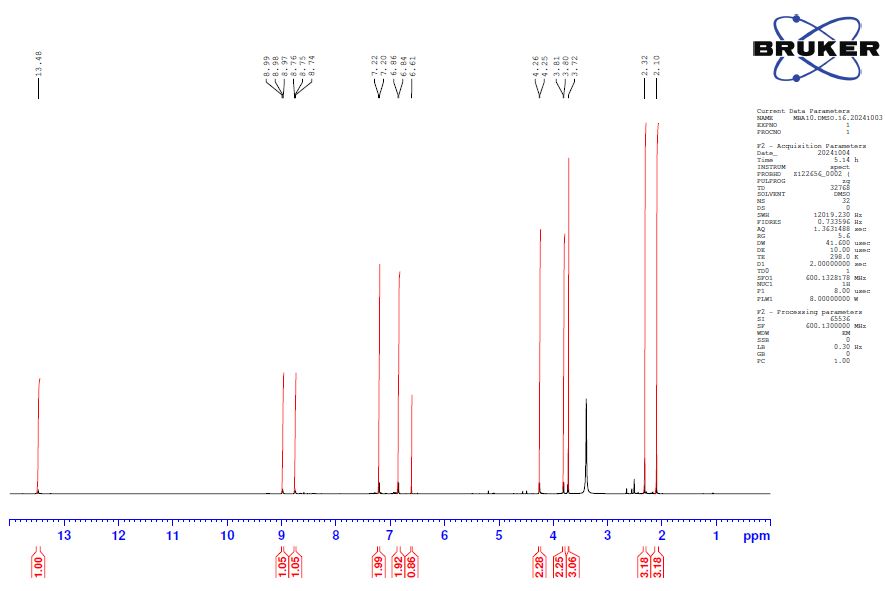^  ^1^H NMR for **3e**  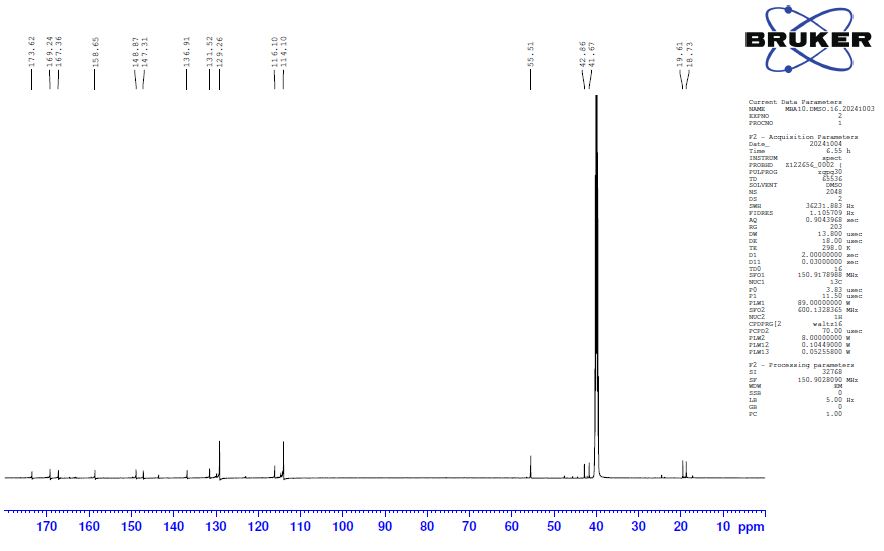  ^1^C NMR for **3e** |
| **3f** | 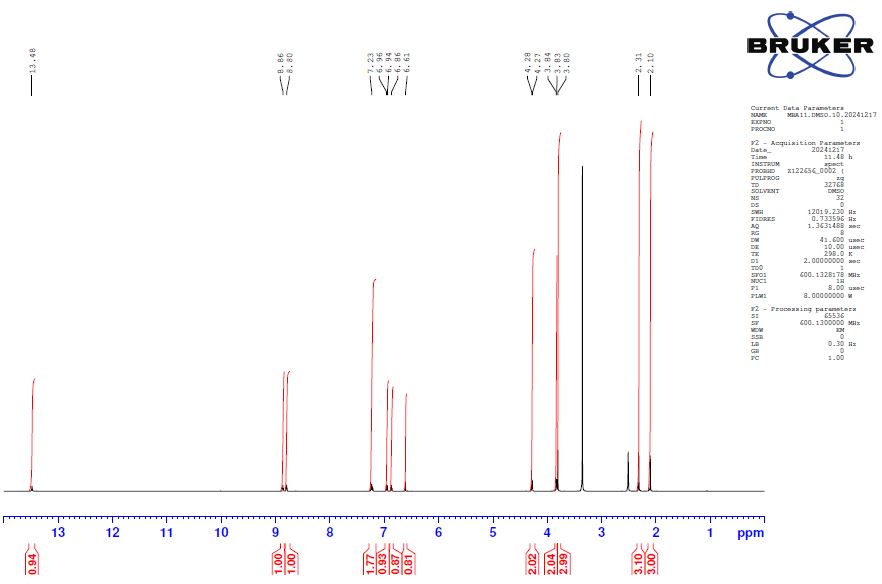  ^1^H NMR for **3f**  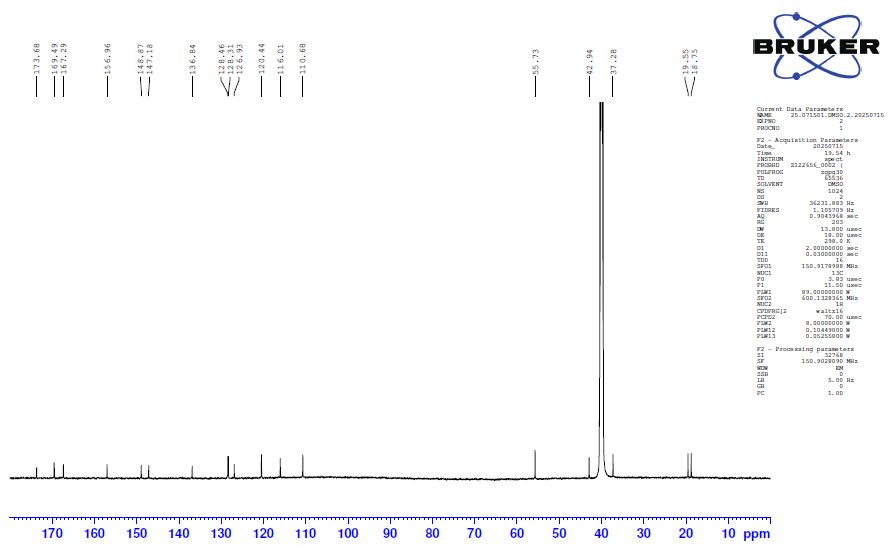  ^1^C NMR for **3f** |
| **3g** | 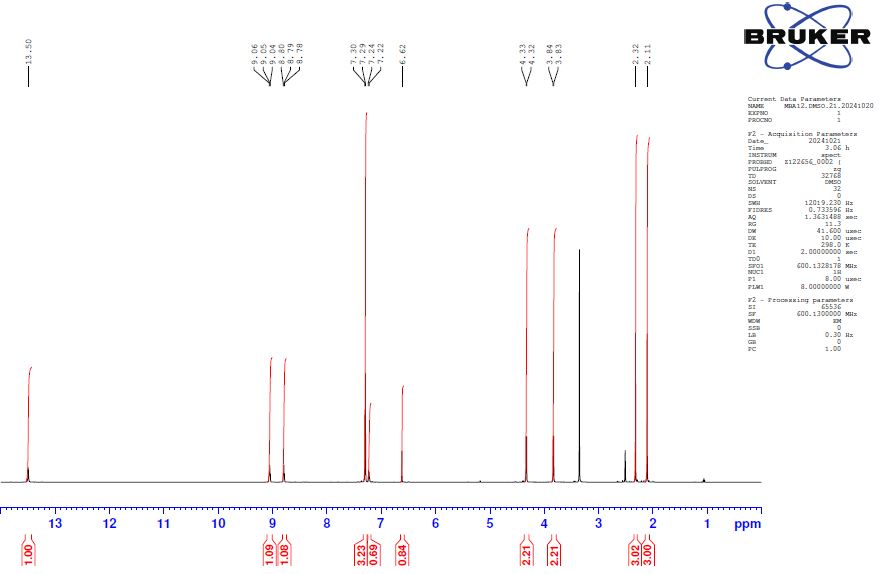  ^1^H NMR for **3g**  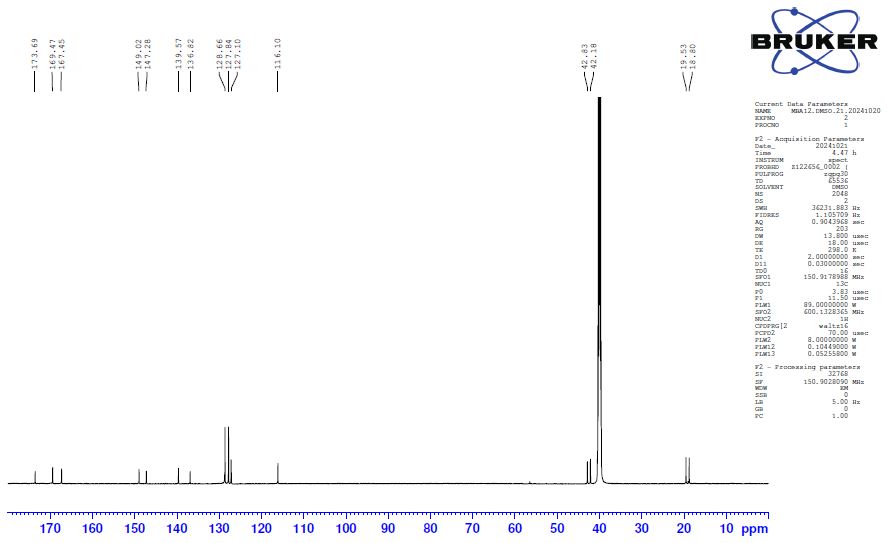  ^1^C NMR for **3g** |
| **3h** | 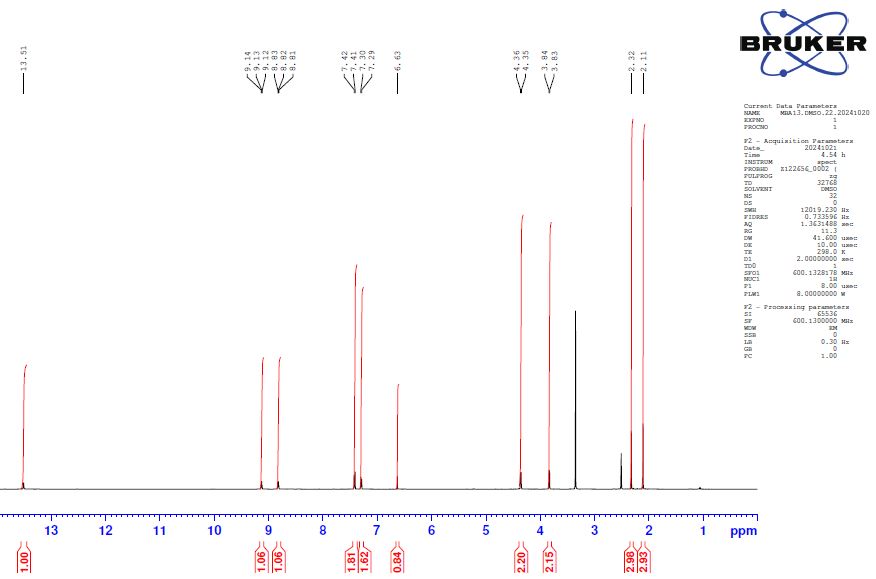  ^1^H NMR for **3h**  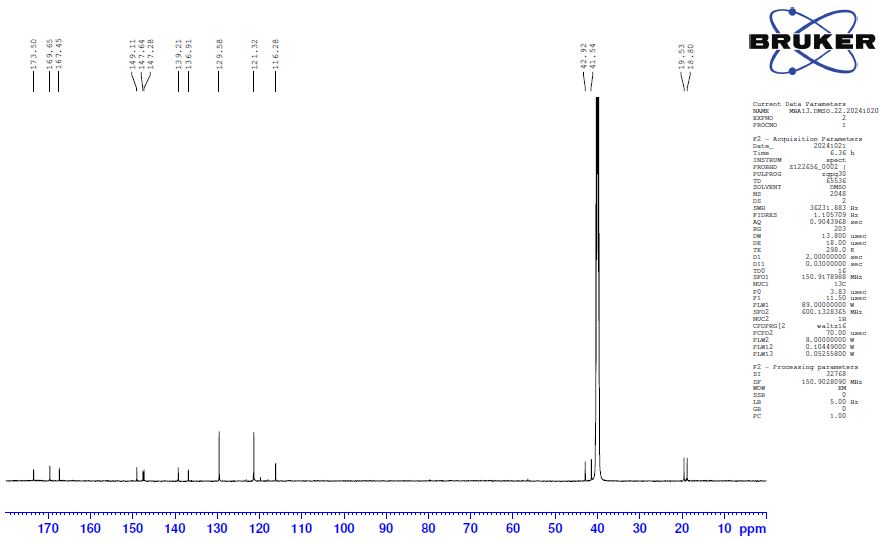  ^1^C NMR for **3h** |
| **3i** | ^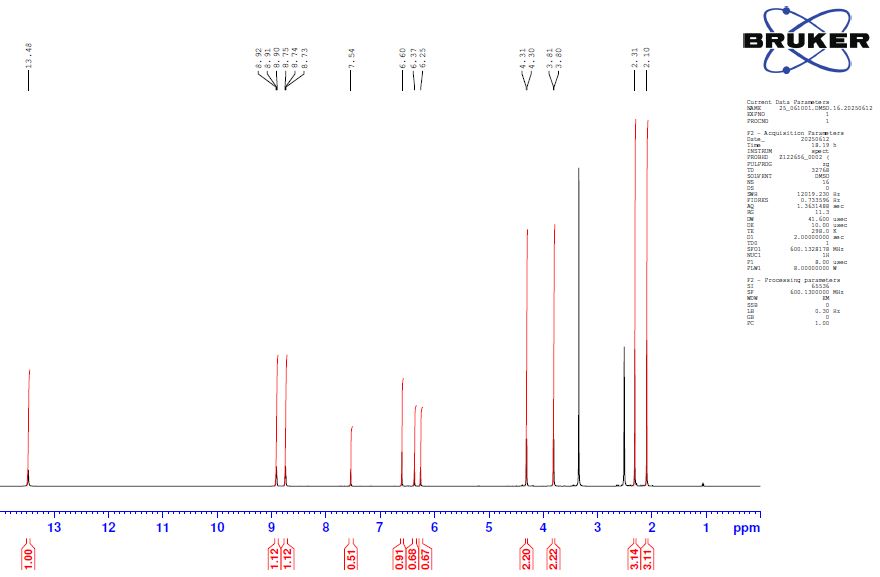^  ^1^H NMR for **3i**  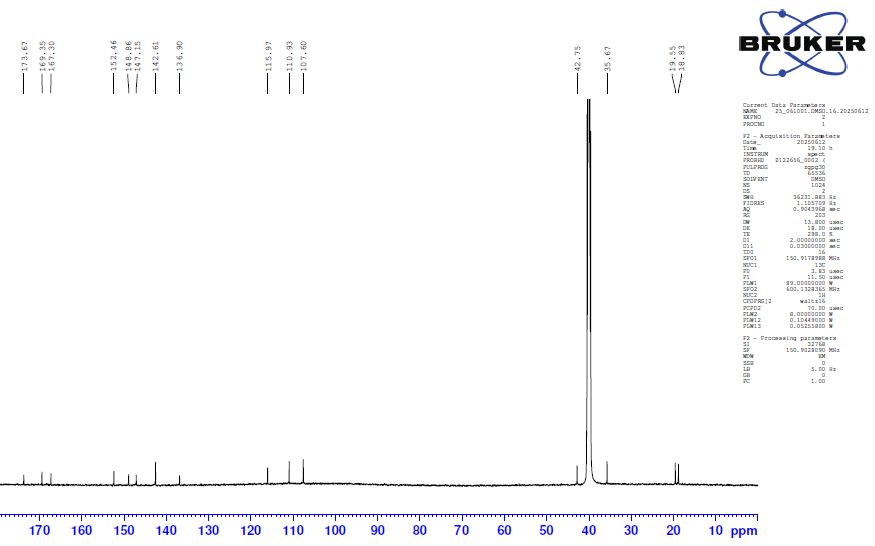  ^1^C NMR for **3i** |
| **3j** | 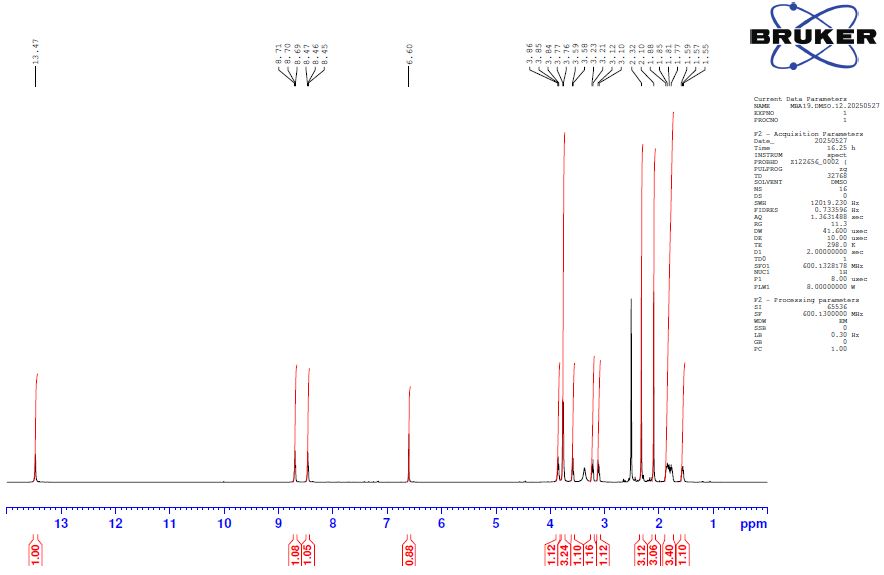  ^1^H NMR for **3j**  ^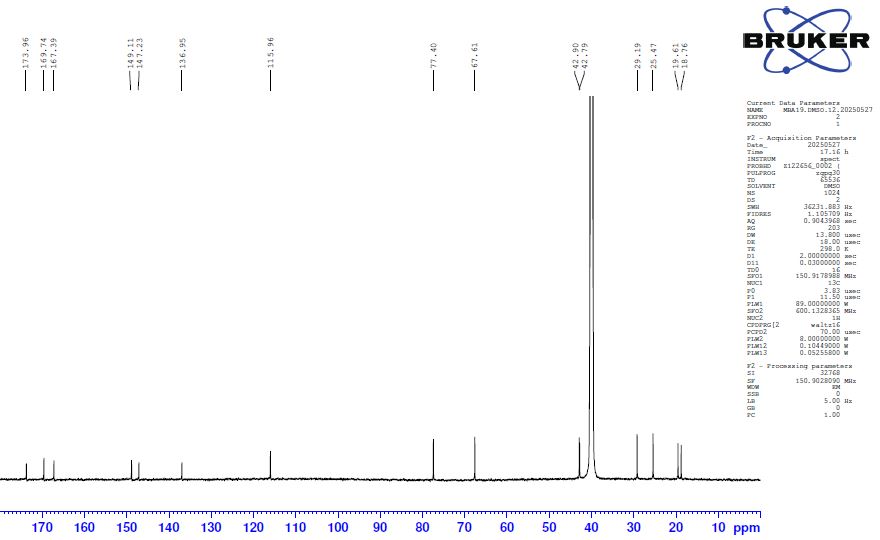^  ^1^C NMR for **3j** |
| **3k** | ^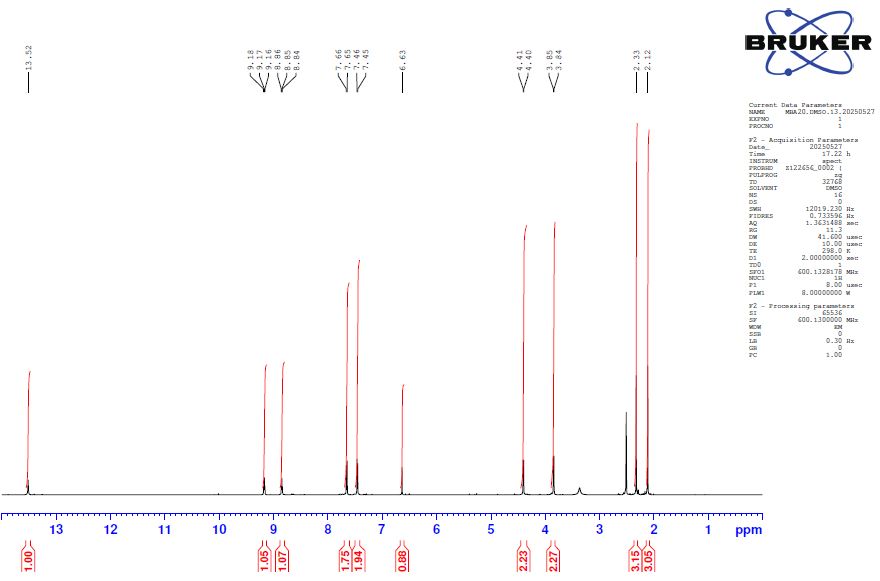^  ^1^H NMR for **3k**  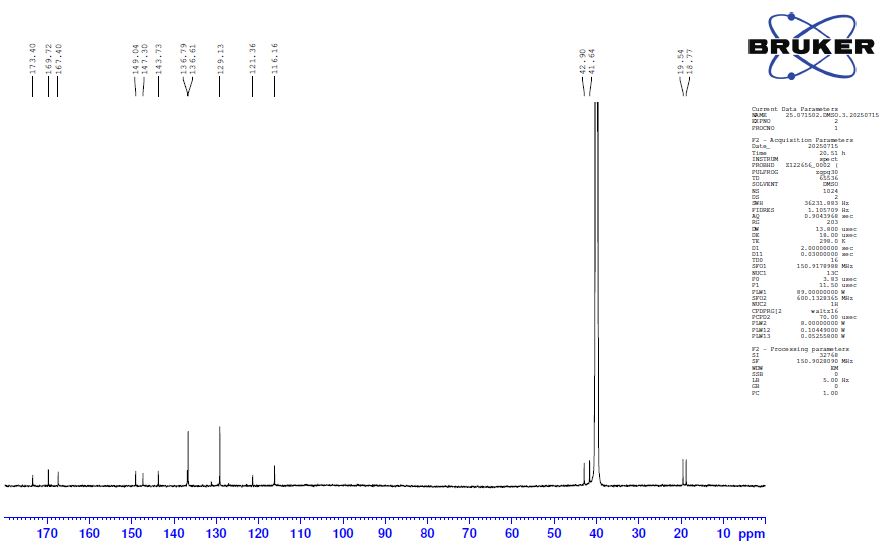  ^1^C NMR for **3k** |

**Table S2**. Visualizations of Fourier-Transform Infrared (FT-IR) spectra of compounds **3a**-**k**.

% Transmittance

| Comp. | ATR-FTIR spectra |
| --- | --- |
| **3a** | 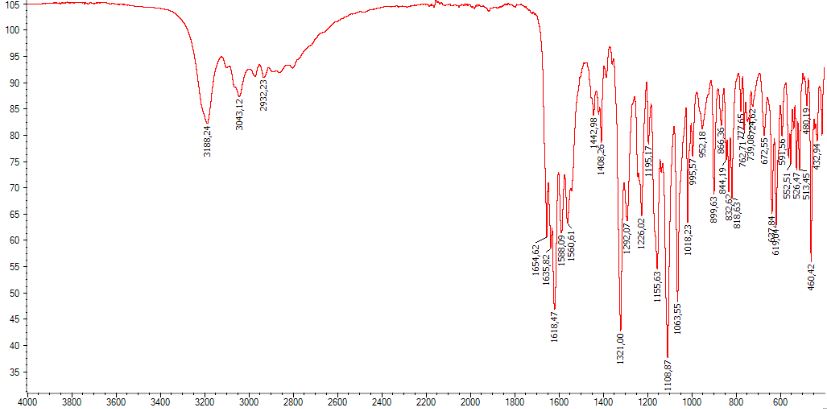  % Wavenumbers (cm^-1^) |
| **3b**  % Transmittance | 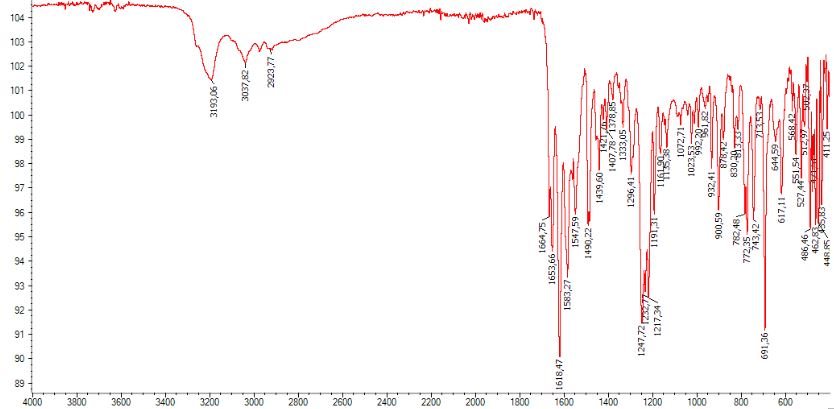  % Wavenumbers (cm^-1^) |
| **3c**  % Transmittance | 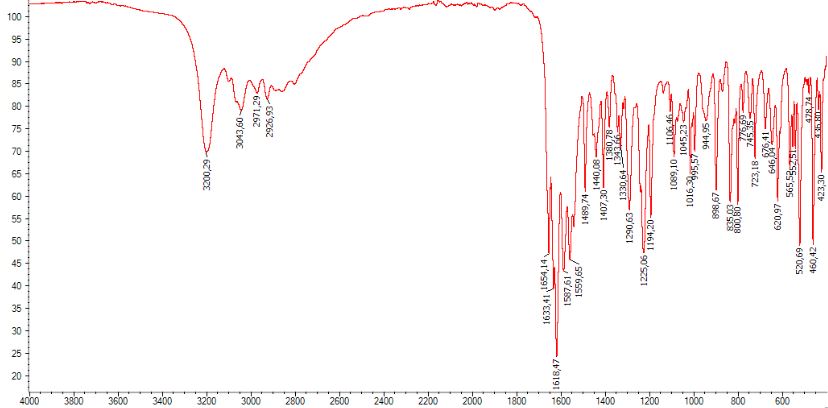  % Wavenumbers (cm^-1^) |
| **3d** | 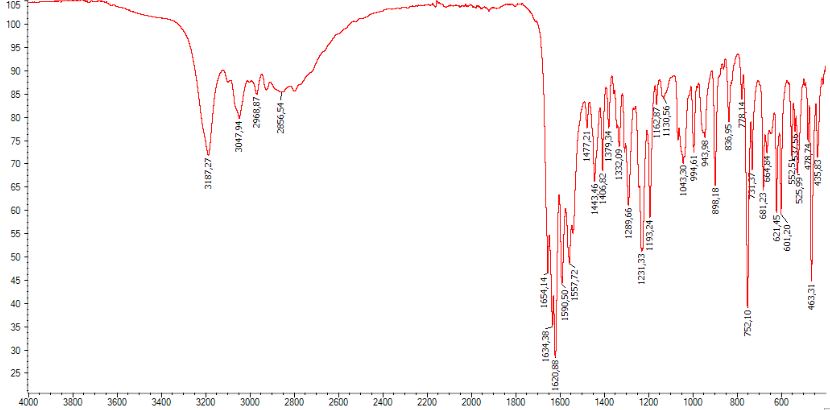  % Wavenumbers (cm^-1^)  % Transmittance |
| **3e**  % Transmittance | 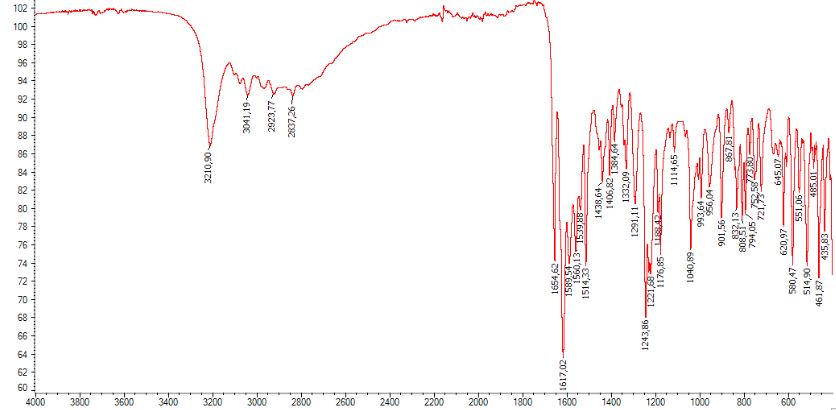  % Wavenumbers (cm^-1^) |
| **3f**  % Transmittance | 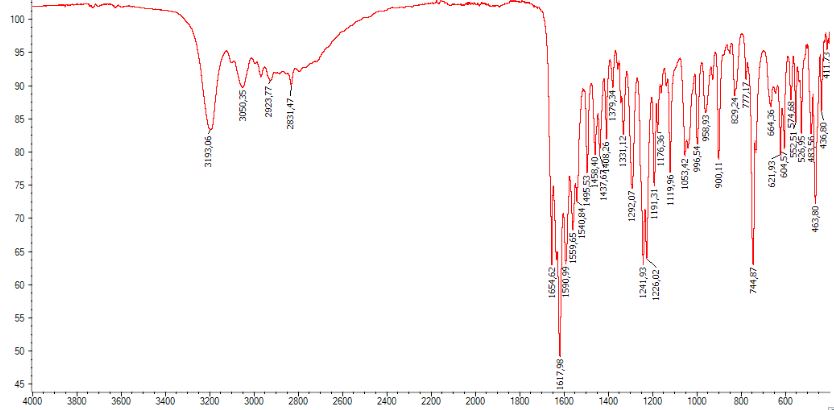  % Wavenumbers (cm^-1^) |
| **3g**  % Transmittance | 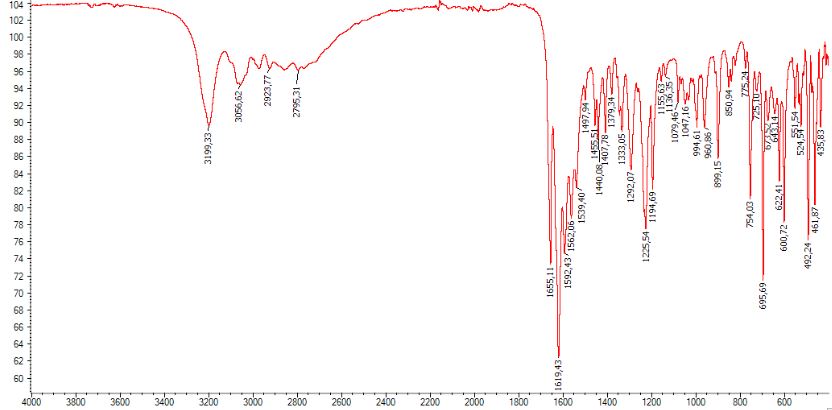  % Wavenumbers (cm^-1^) |
| **3h**  % Transmittance | 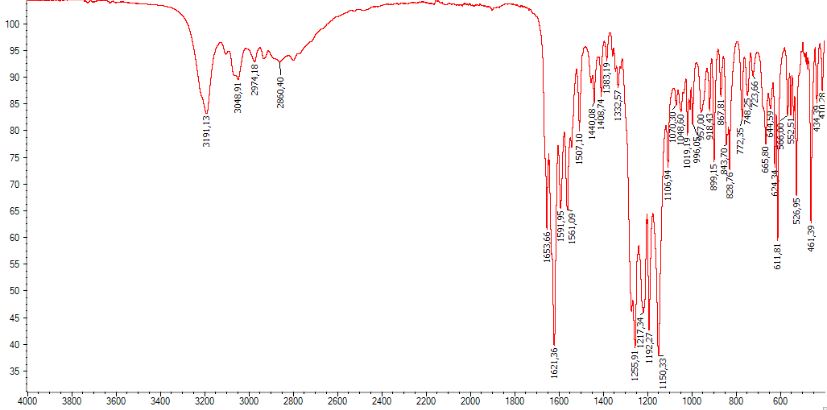  % Wavenumbers (cm^-1^) |
| **3i**  % Transmittance | 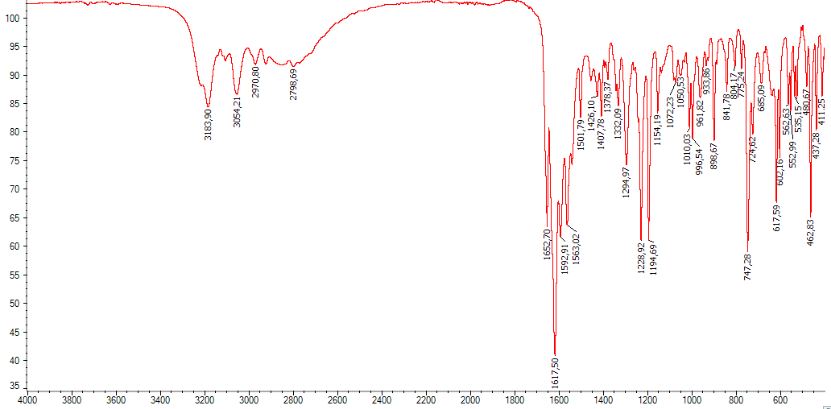  % Wavenumbers (cm^-1^) |
| **3j** | 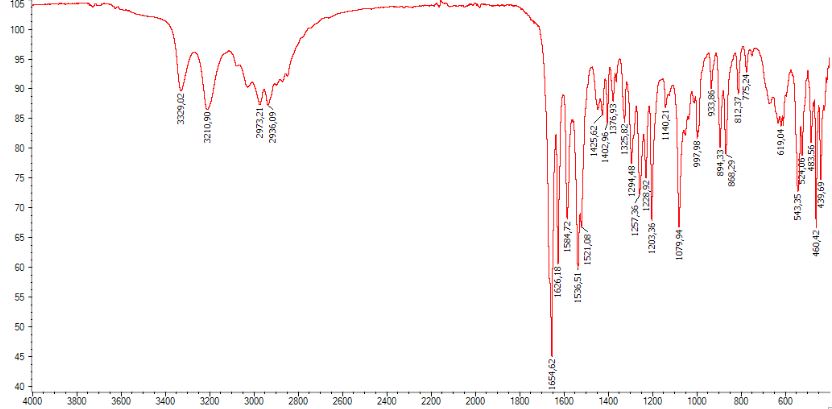  % Wavenumbers (cm^-1^)  % Transmittance |
| **3k**  % Transmittance | 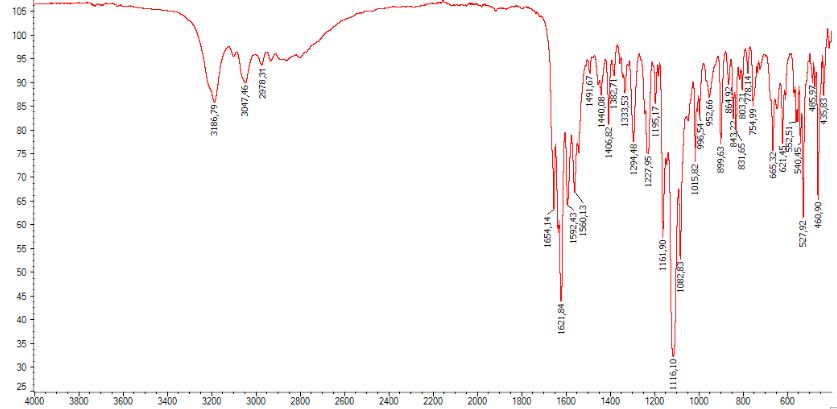  % Wavenumbers (cm^-1^) |

**Table S3.** Visualizations of High Resolution Mass Spectrometry (HRMS) spectra of compounds **3a**-**k**.

| Comp. | HRMS spectra | |
| --- | --- | --- |
| **3a** |  |  |
| **3b** |  |  |
| **3c** |  |  |
| **3d** |  |  |
| **3e** |  |  |
| **3f** |  |  |
| **3g** |  |  |
| **3h** |  |  |
| **3i** |  |  |
| **3j** |  |  |
| **3k** |  |  |

| **Table S4**. HOMO and LUMO molecular orbitals of compounds 3a-k. | | |
| --- | --- | --- |
| Compound: | HOMO | LUMO |
| **3a** | 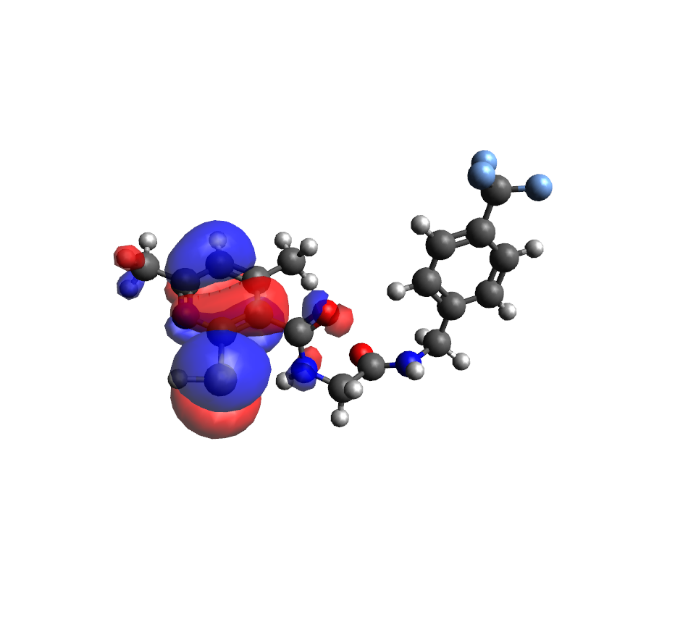 | 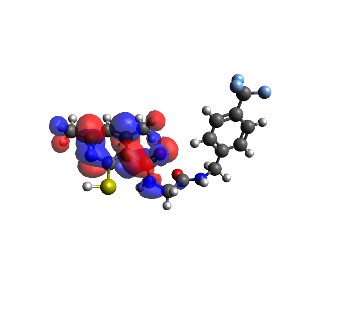 |
| **3b** | 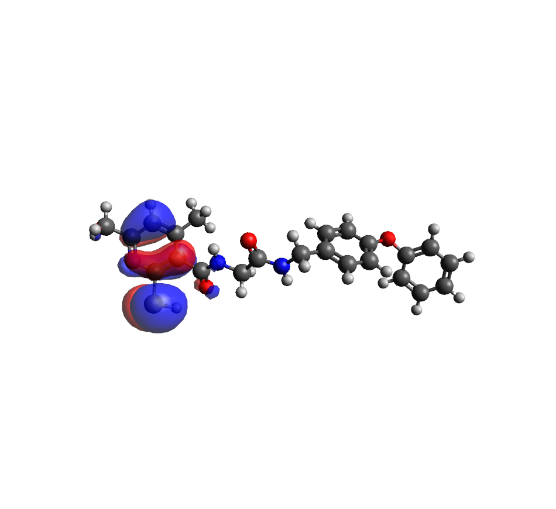 | 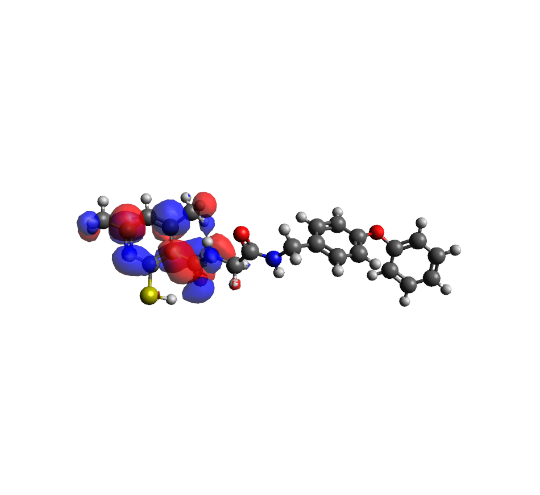 |
| **3c** | 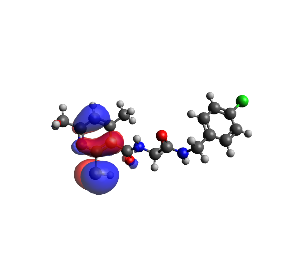 | 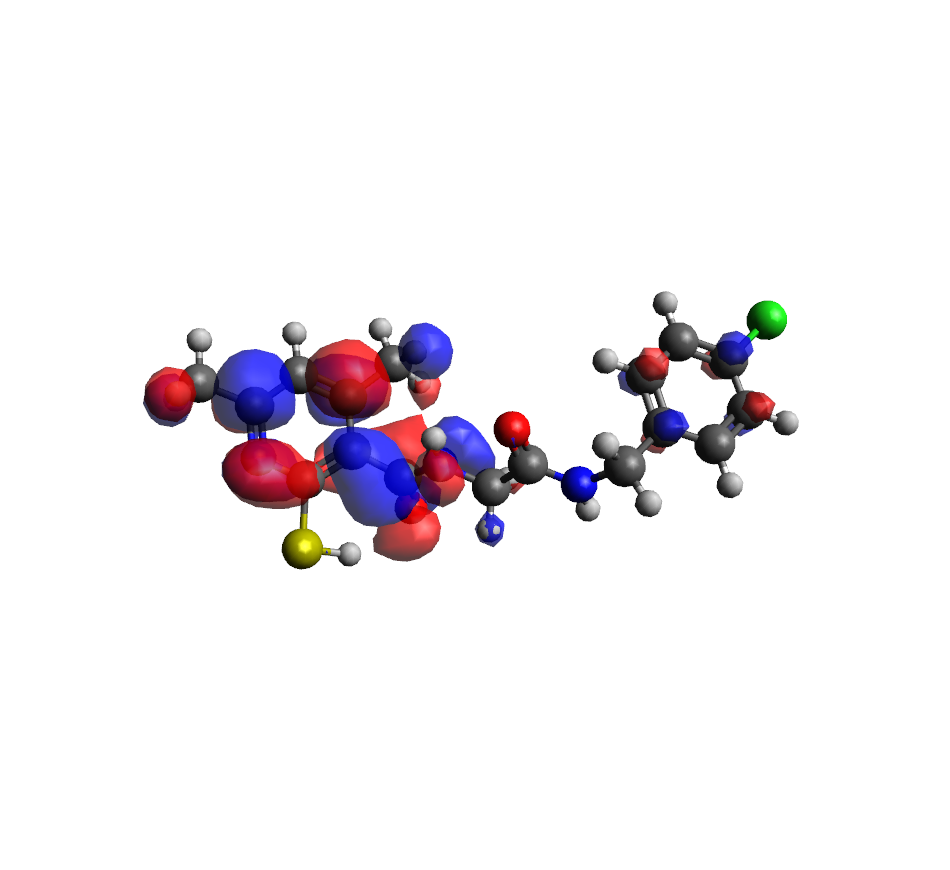 |
| **3d** | 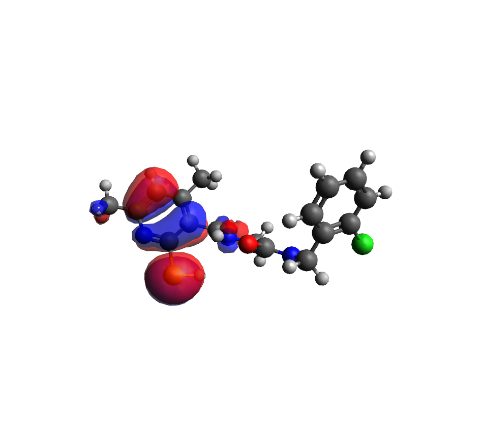 | 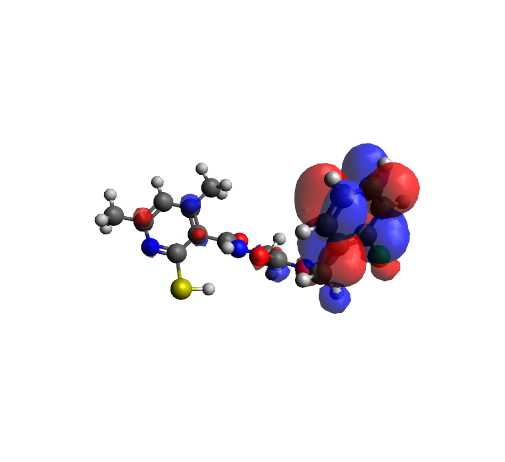 |
| **3e** | 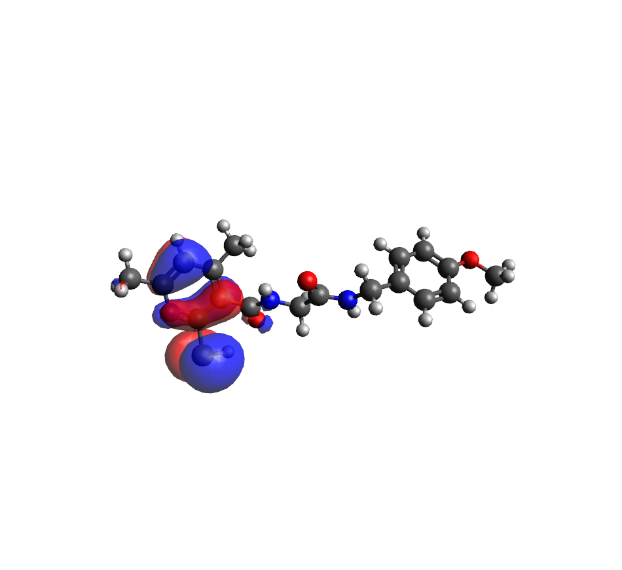 | 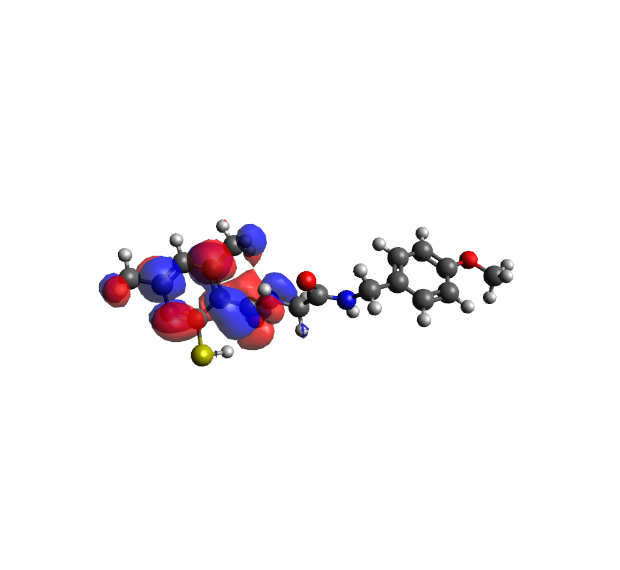 |
| **3f** | 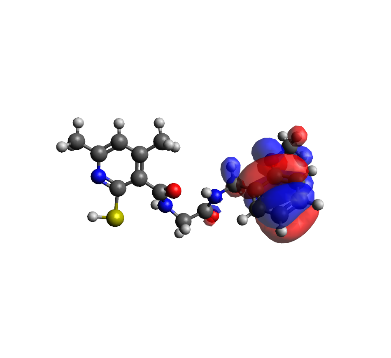 | 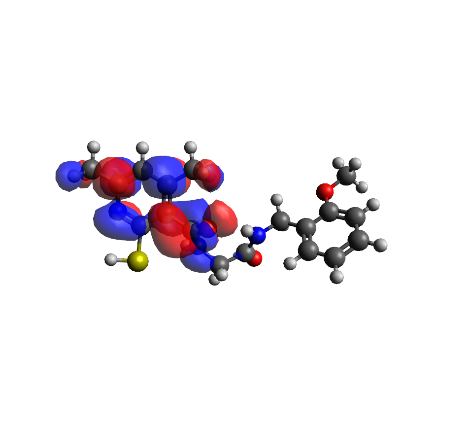 |
| **3g** | 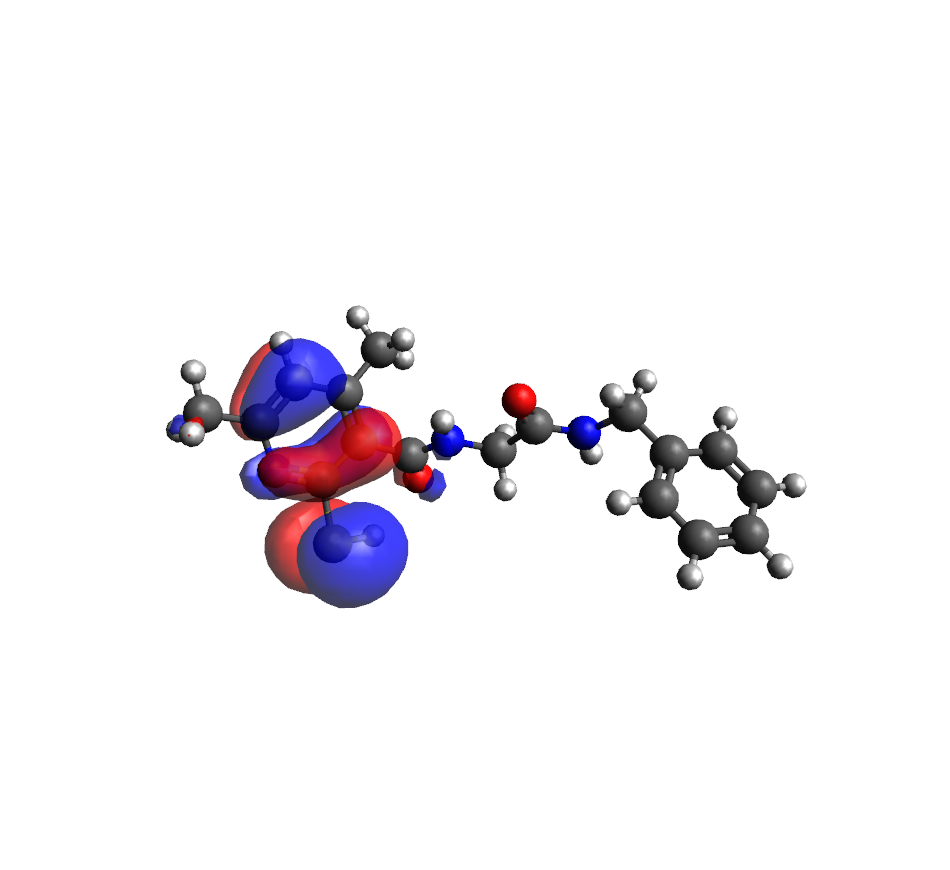 | 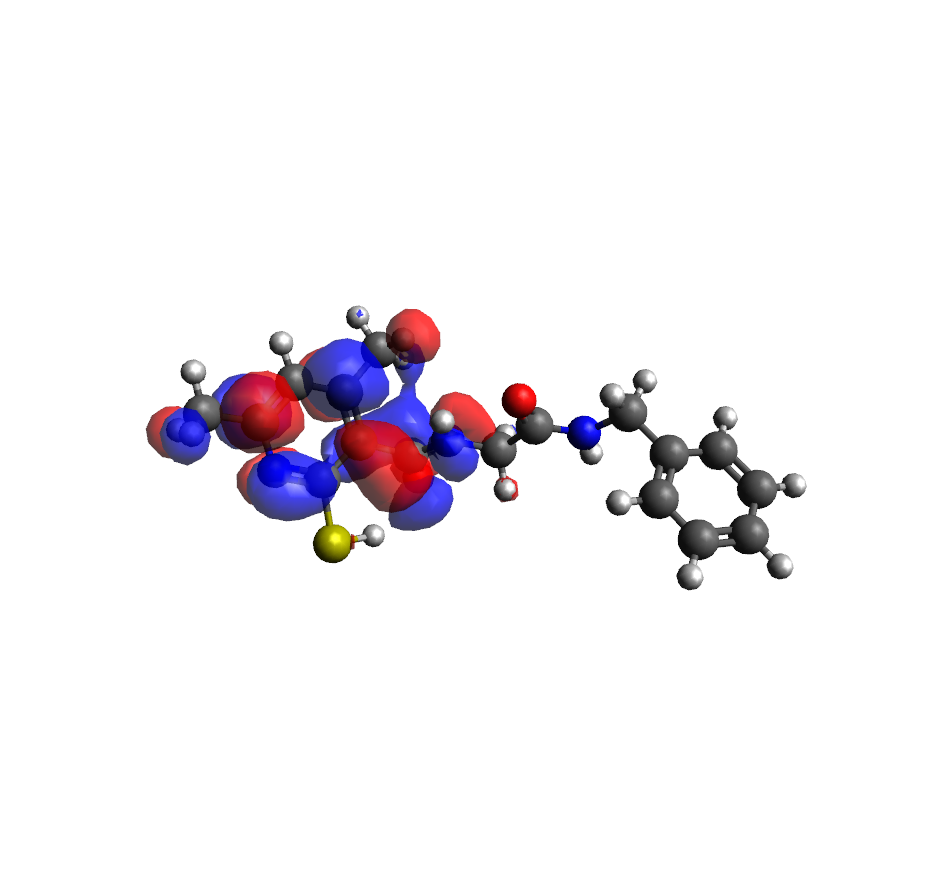 |
| **3h** | 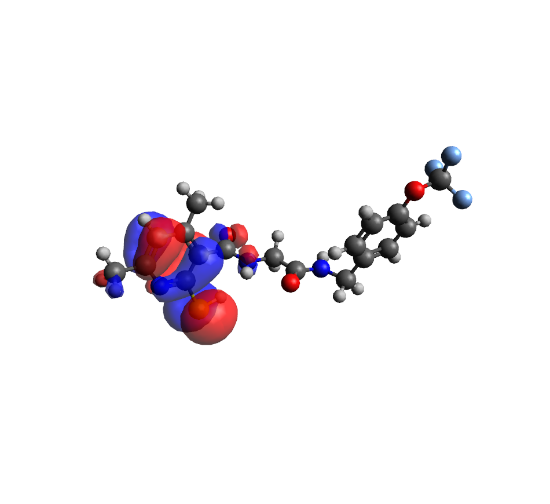 | 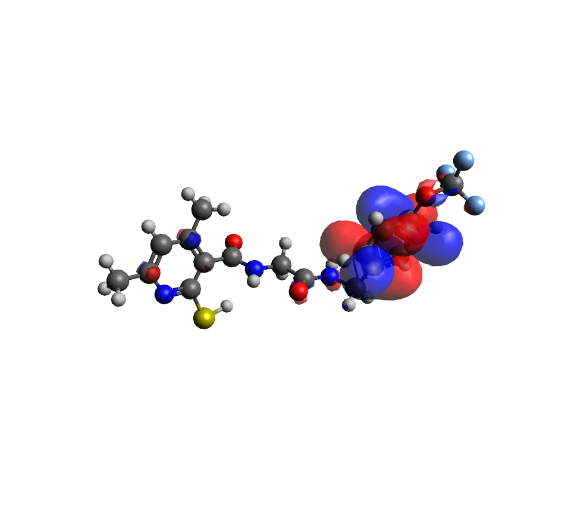 |
| **3i** | 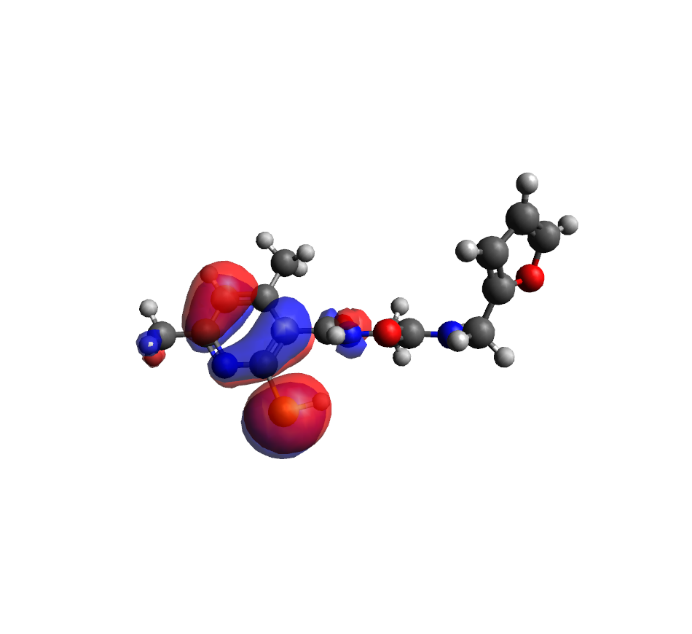 | 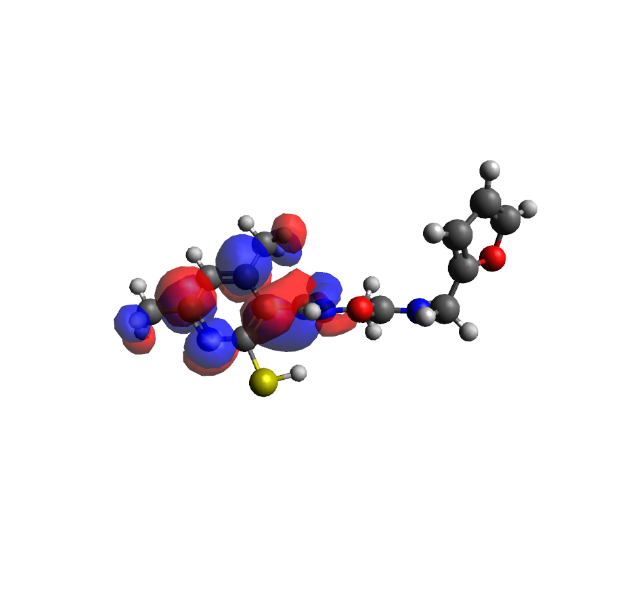 |
| **3j** | 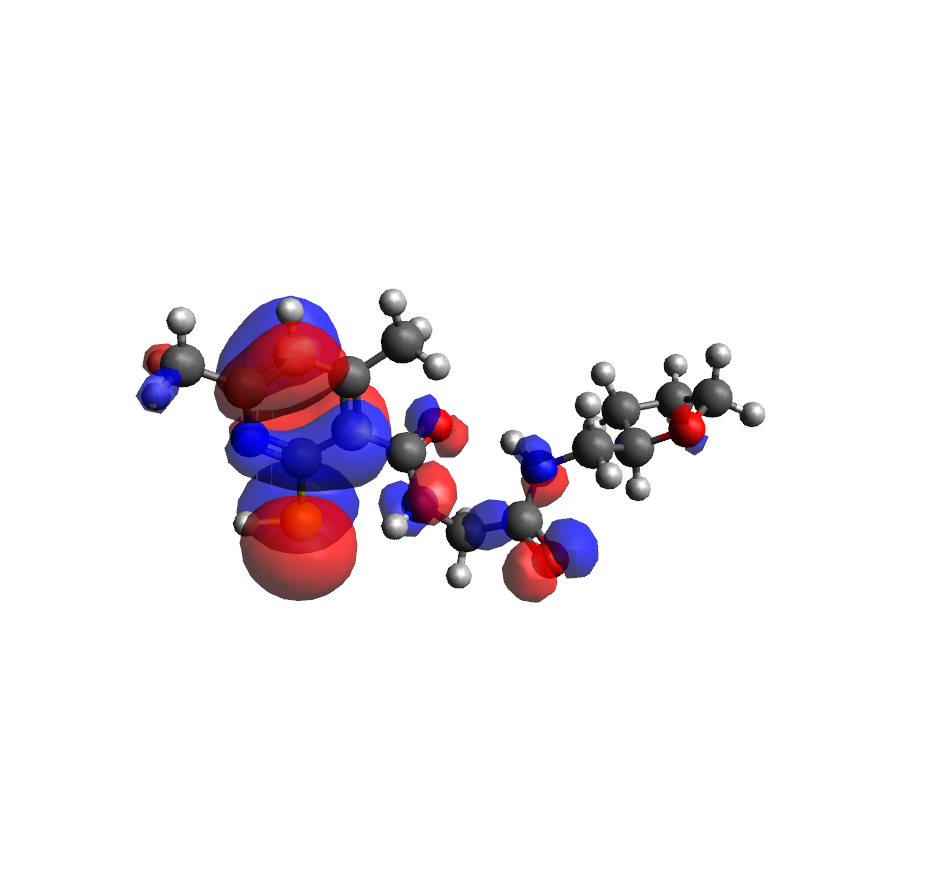 | 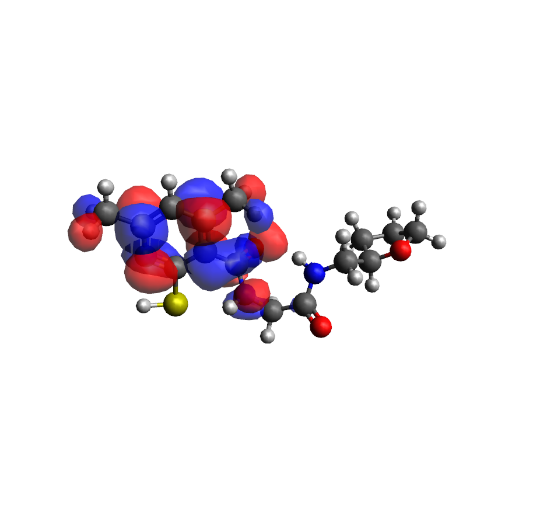 |
| **3k** | 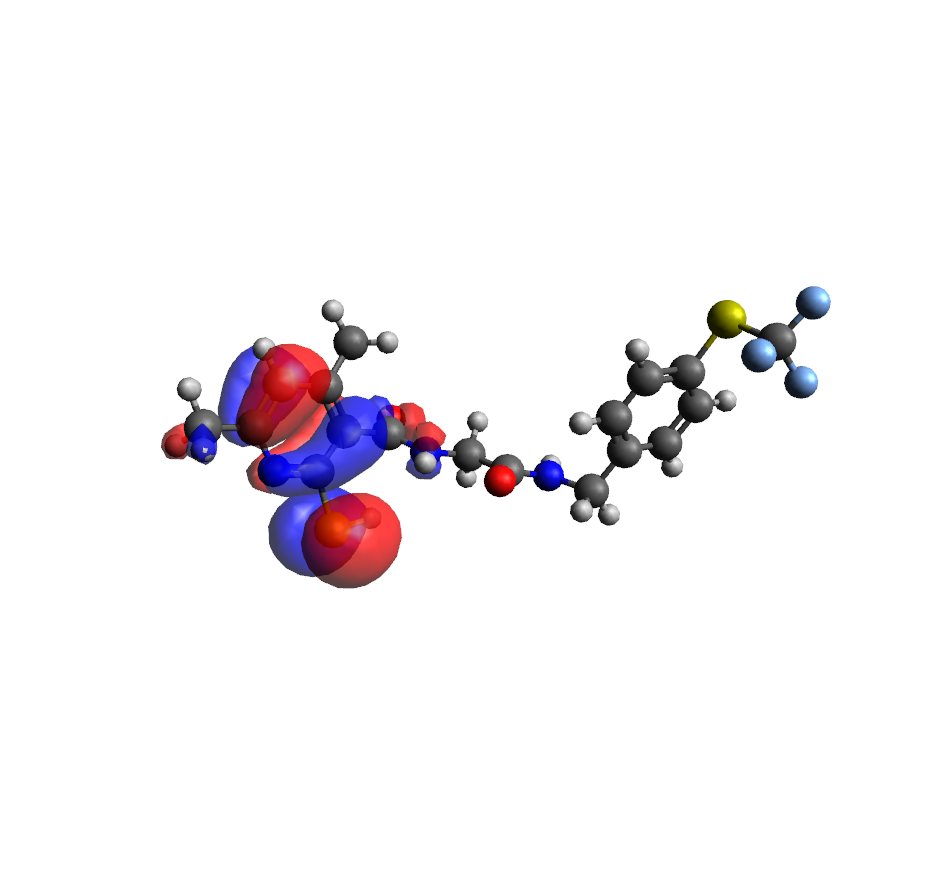 | 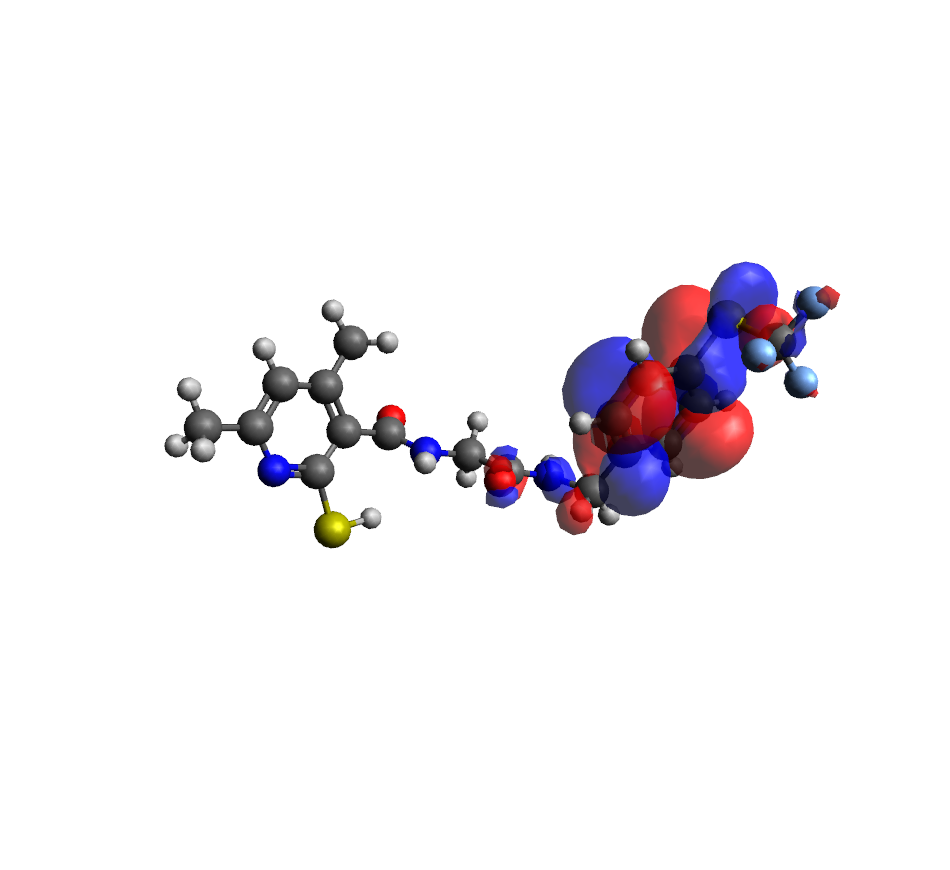 |

| **Table S5**. 2D interaction diagrams between the designed compounds and MMP-13 and MMP-8. | | |
| --- | --- | --- |
| **Compound:** | **MMP-13** | **MMP-8** |
| **3a** | **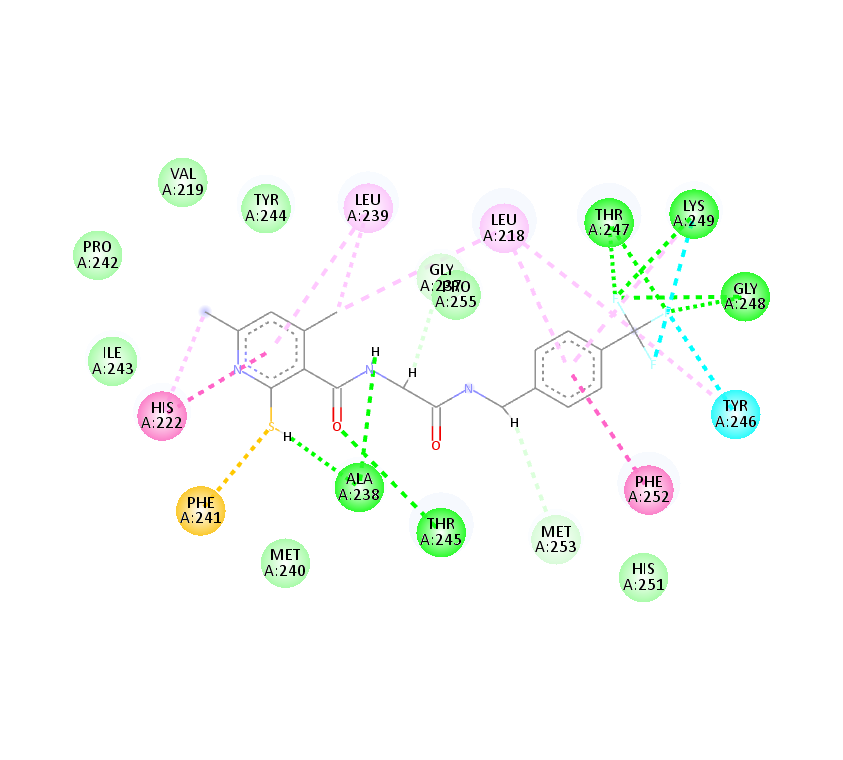** | **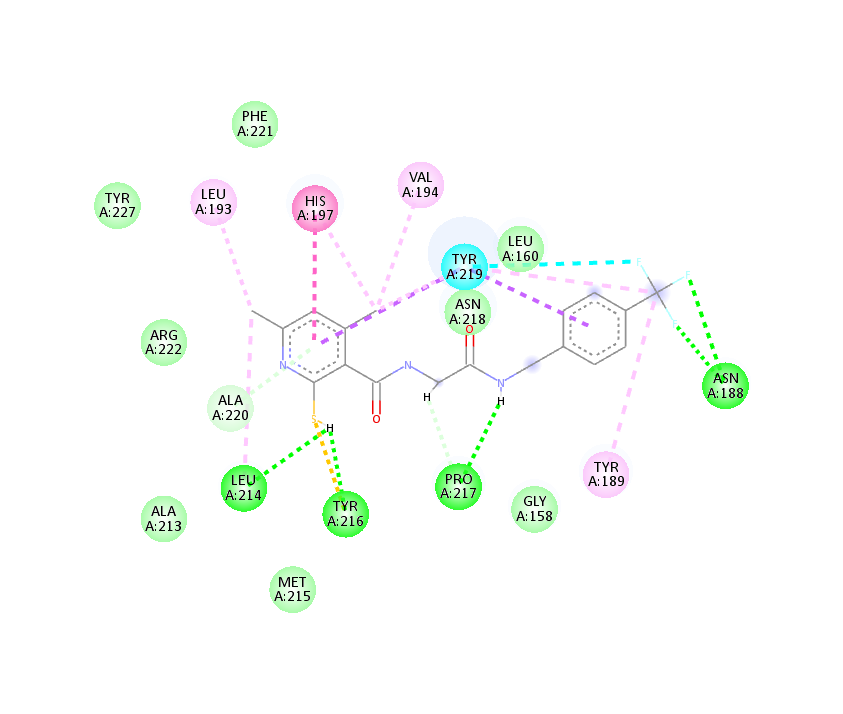** |
| **3e** | **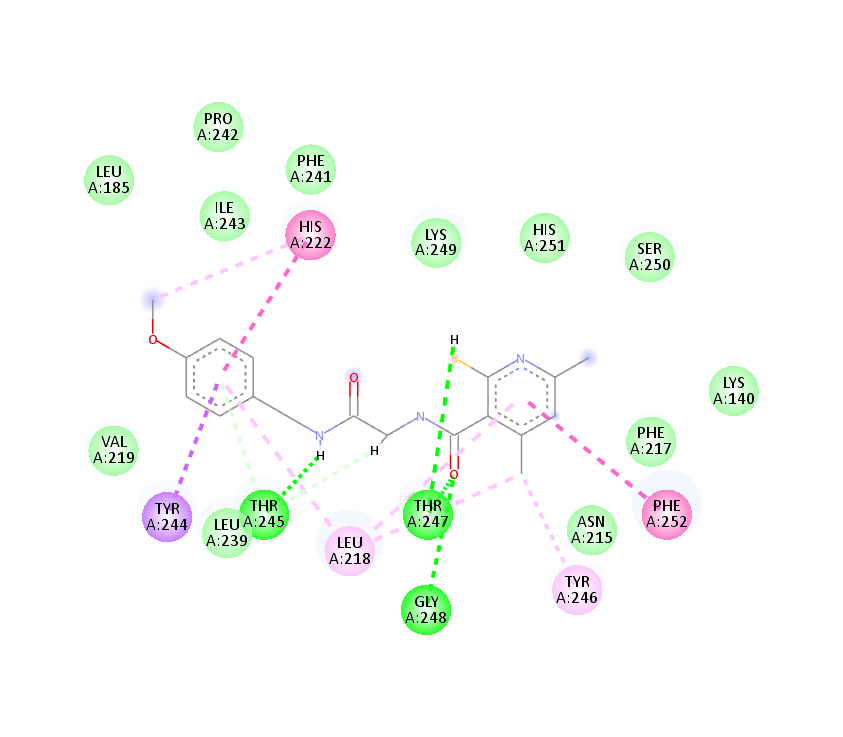** | **6**  **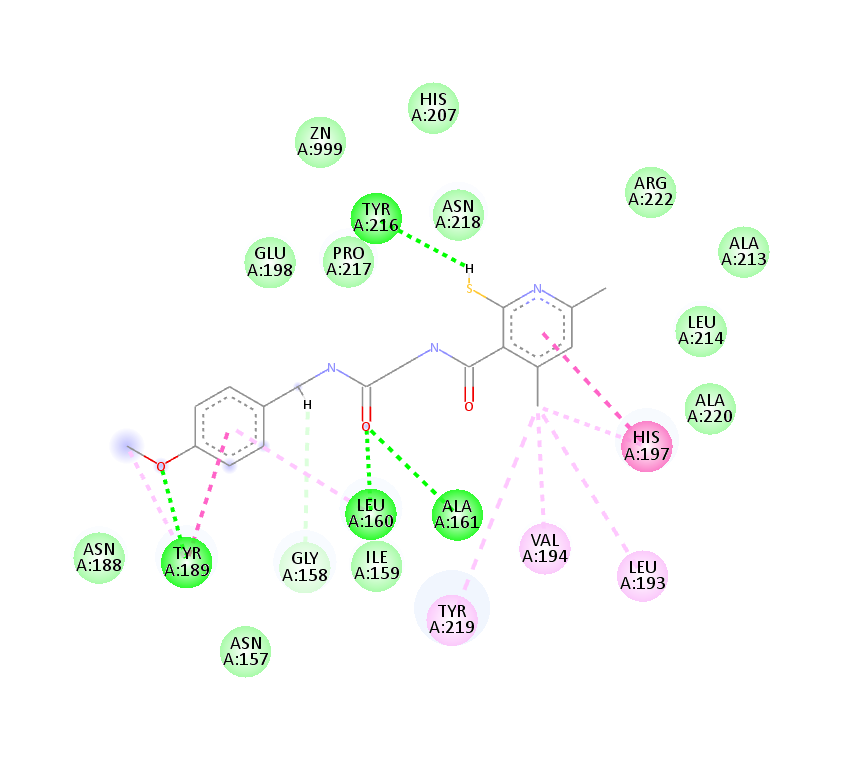** |
| **3f** | **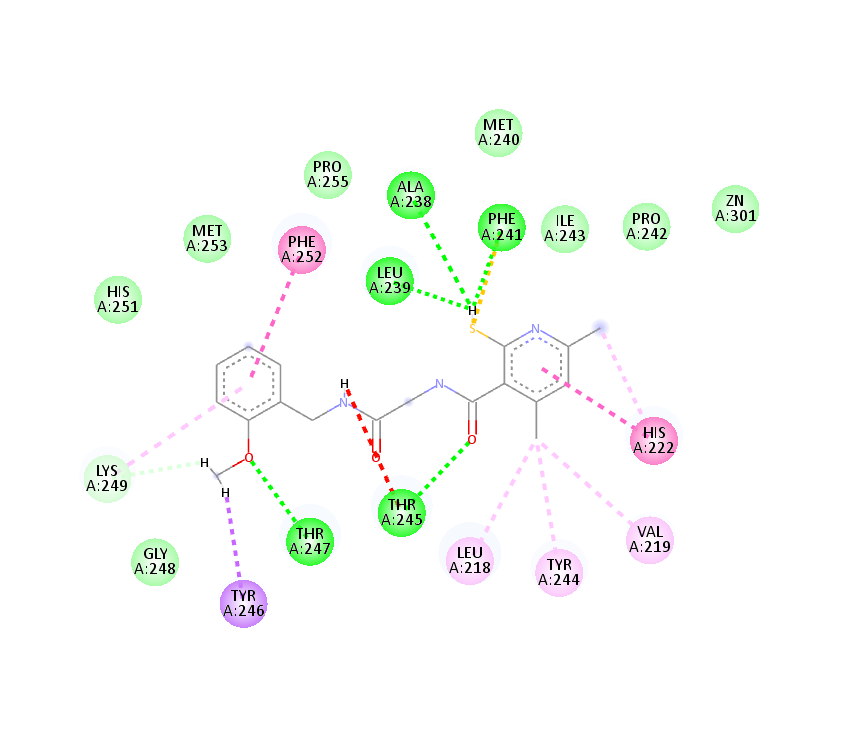** | **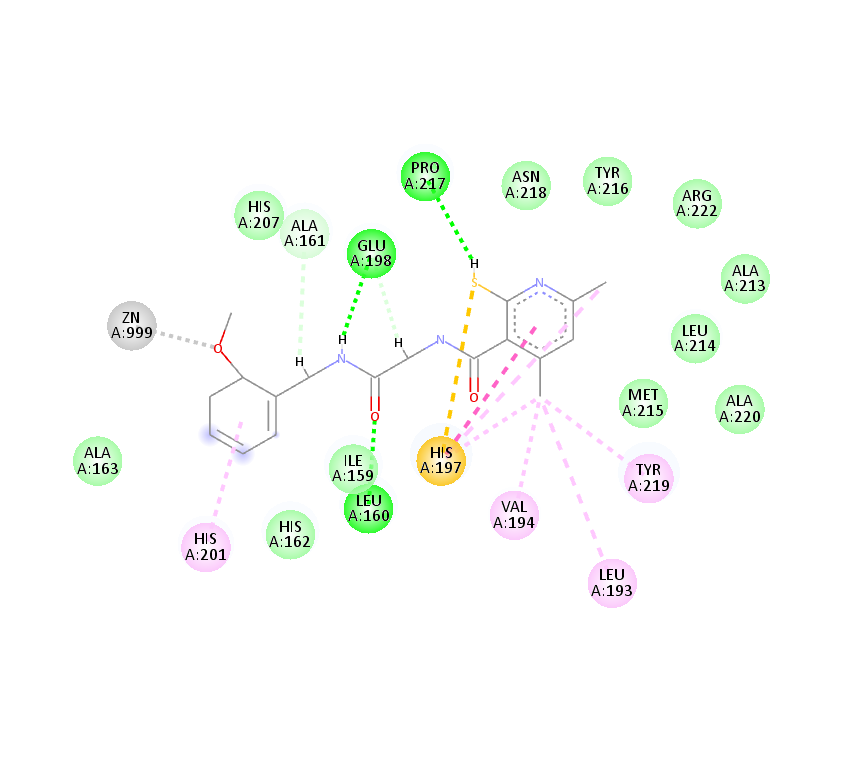** |
| **3h** | **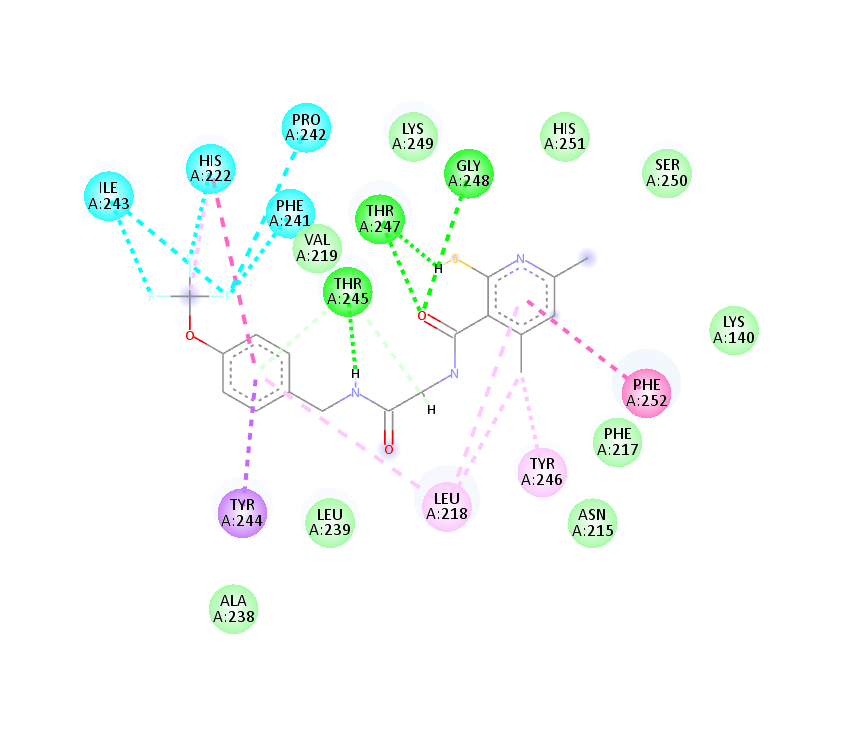** | **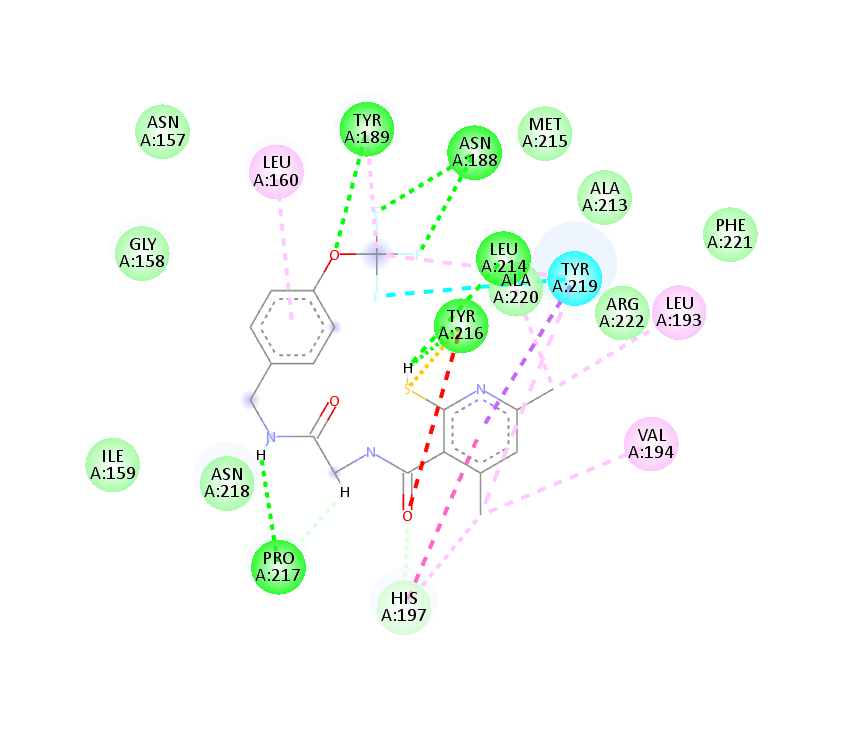** |
| **3i** | **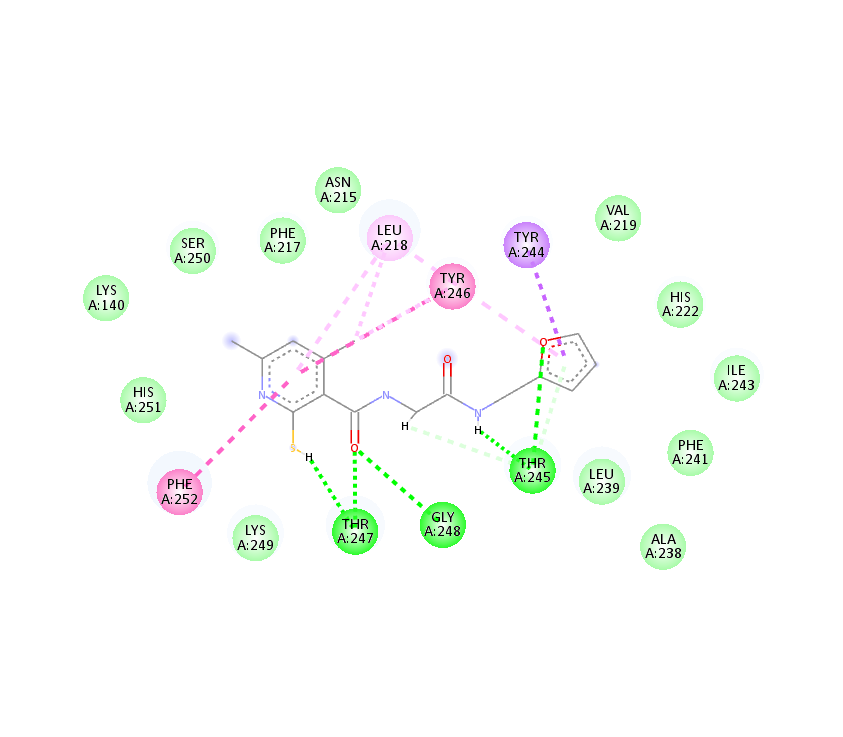** | **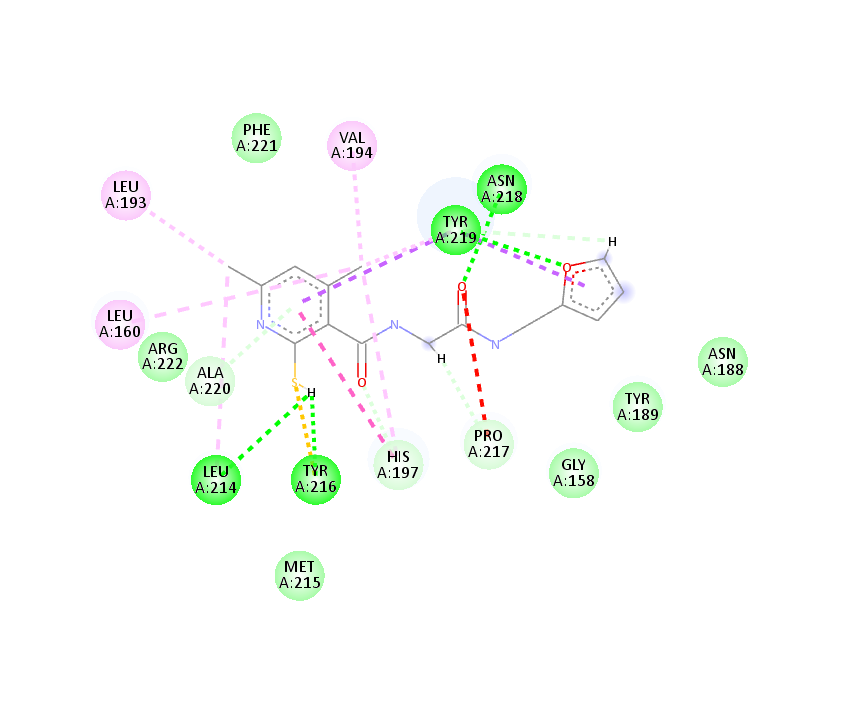** |
| **3j** | **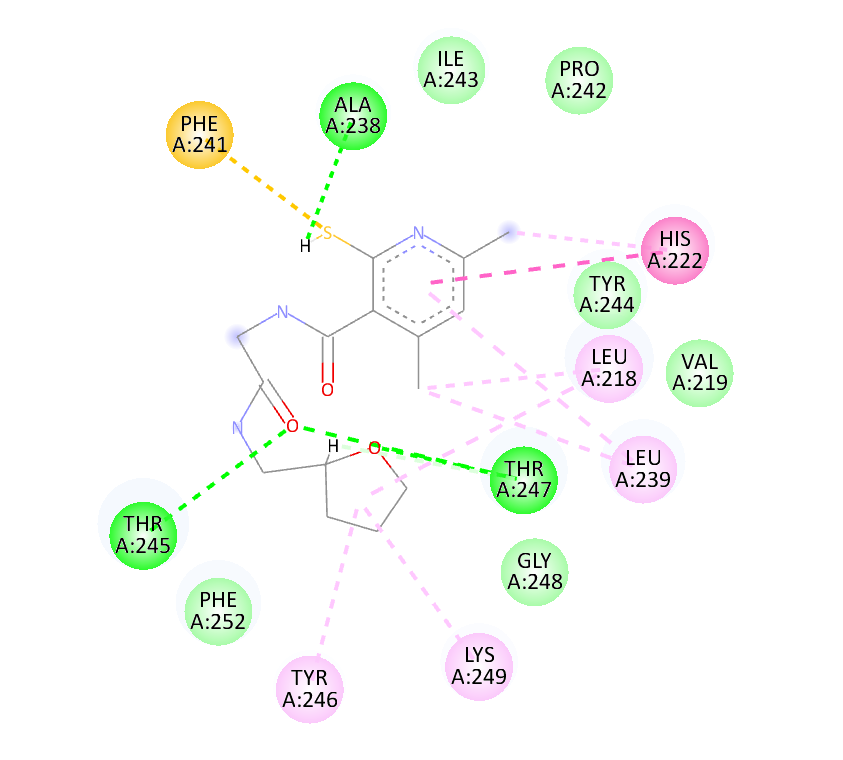** | **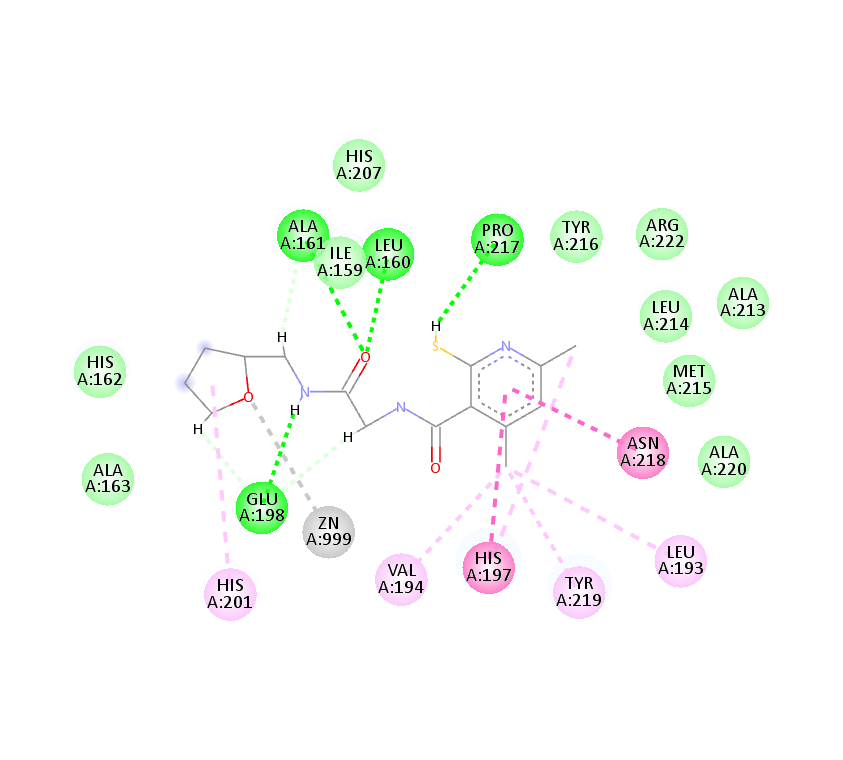** |
| **3k** | **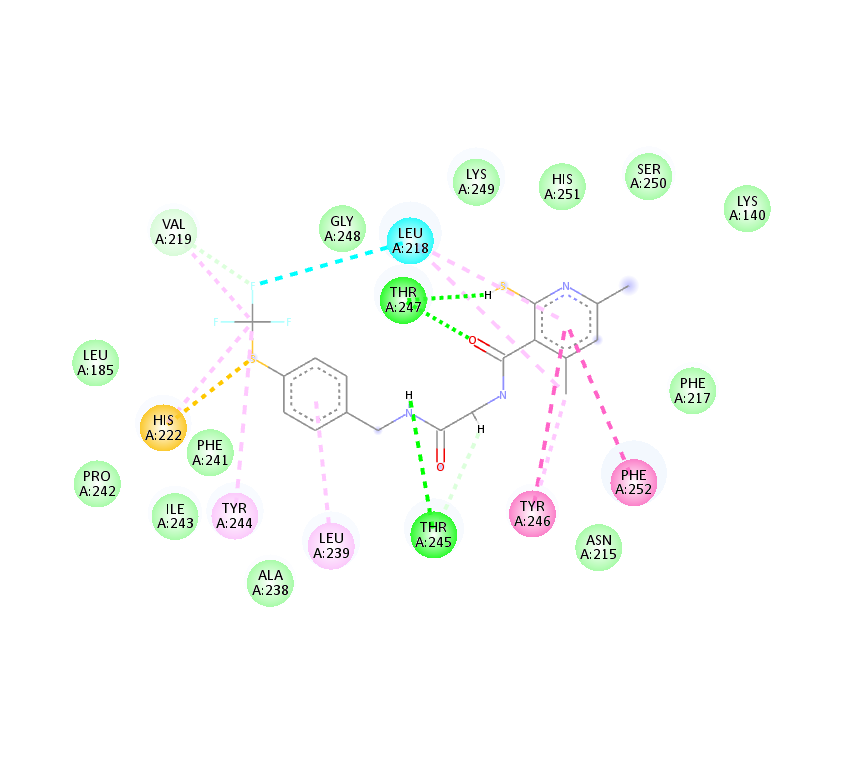** | **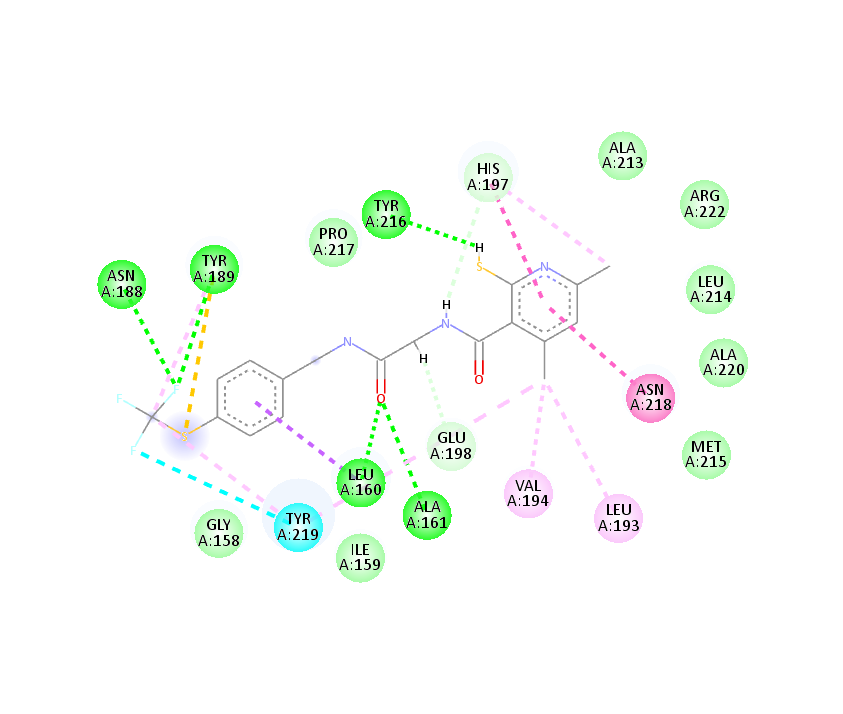** |
| **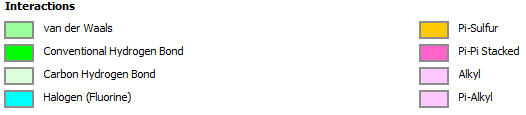** | | |

| **A** | **B** |
| --- | --- |
| **C**   | **D**   |
| **Figure S1**. The Stearn-Volmer plots Double logarithm regression plot of the fluorescence quenching of HSA by compounds: A - 3b, B - 3c, C - 3d, and D - 3g. | |

| **A** | **B** |
| --- | --- |
| **C** | **D** |
| **Figure S2**. Double logarithm regression plot of the fluorescence quenching of HSA by compounds: A - 3b, B - 3c, C - 3d, and D - 3g. | |

| **Table S6.** ADMET properties of compounds 3a-3k. | | | | | | | |
| --- | --- | --- | --- | --- | --- | --- | --- |
| **Properties:** | **3a** | **3e** | **3f** | **3h** | **3i** | **3j** | **3k** |
| **Lipinski Rule** | Accepted | Accepted | Accepted | Accepted | Accepted | Accepted | Accepted |
| **TPSA** | 71.09 | 80.32 | 80.32 | 80.32 | 84.23 | 80.32 | 71.09 |
| **Absorption** | | | | | | | |
| **Caco-2 Permeability** | -5.26 | -5.48 | -5.56 | -4.87 | -5.21 | -5.51 | -5.06 |
| **MDCK Permeability** | -4.85 | -5.06 | -4.95 | -4.73 | -4.82 | -4.99 | -4.79 |
| **HIA** | > 30% | > 30% | > 30% | > 30% | > 30% | > 30% | > 30% |
| **Distribution** | | | | | | | |
| **PPB** | 98.80% | 97.80% | 97.80% | 98.80% | 97.90 % | 93.30% | 99.00% |
| **VD_ss_ [L/kg]** | 1.63 | 1.16 | 1.26 | 1.10 | 1.01 | 1.40 | 1.25 |
| **Excretion** | | | | | | | |
| **CL_plasma_ [mL/min/kg]** | 5.80 | 5.48 | 5.04 | 4.74 | 4.71 | 5.01 | 4.86 |
| **T_1/2_ [h]** | 0.52 | 0.46 | 0.47 | 0.50 | 0.52 | 0.60 | 0.57 |
| **Toxicity** *The output value is the probability of being toxic within the range of 0 to 1.* | | | | | | | |
| **hERG Blockers** | 0.05 | 0.04 | 0.03 | 0.17 | 0.02 | 0.08 | 0.06 |
| **AMES Toxicity** | 0.42 | 0.76 | 0.64 | 0.33 | 0.64 | 0.61 | 0.51 |
| **Rat Oral Acute Toxicity** | 0.50 | 0.38 | 0.41 | 0.68 | 0.57 | 0.43 | 0.49 |
| **Carcinogenicity** | 0.07 | 0.24 | 0.20 | 0.11 | 0.36 | 0.28 | 0.08 |
